# Models of *KPTN*-related disorder implicate mTOR signalling in cognitive and overgrowth phenotypes

**DOI:** 10.1093/brain/awad231

**Published:** 2023-07-12

**Authors:** Maria O Levitin, Lettie E Rawlins, Gabriela Sanchez-Andrade, Osama A Arshad, Stephan C Collins, Stephen J Sawiak, Phillip H Iffland, Malin H L Andersson, Caleb Bupp, Emma L Cambridge, Eve L Coomber, Ian Ellis, Johanna C Herkert, Holly Ironfield, Logan Jory, Perrine F Kretz, Sarina G Kant, Alexandra Neaverson, Esther Nibbeling, Christine Rowley, Emily Relton, Mark Sanderson, Ethan M Scott, Helen Stewart, Andrew Y Shuen, John Schreiber, Liz Tuck, James Tonks, Thorkild Terkelsen, Conny van Ravenswaaij-Arts, Pradeep Vasudevan, Olivia Wenger, Michael Wright, Andrew Day, Adam Hunter, Minal Patel, Christopher J Lelliott, Peter B Crino, Binnaz Yalcin, Andrew H Crosby, Emma L Baple, Darren W Logan, Matthew E Hurles, Sebastian S Gerety

**Affiliations:** Wellcome Sanger Institute, Wellcome Genome Campus, Hinxton, Cambridge CB10 1SA, UK; Evox Therapeutics Limited, Oxford OX4 4HG, UK; RILD Wellcome Wolfson Medical Research Centre, University of Exeter, Exeter EX2 5DW, UK; Peninsula Clinical Genetics Service, Royal Devon University Healthcare NHS Foundation Trust, Exeter EX1 2ED, UK; Wellcome Sanger Institute, Wellcome Genome Campus, Hinxton, Cambridge CB10 1SA, UK; Wellcome Sanger Institute, Wellcome Genome Campus, Hinxton, Cambridge CB10 1SA, UK; INSERM Unit 1231, Université de Bourgogne Franche-Comté, Dijon 21078, France; Behavioural and Clinical Neuroscience Institute, University of Cambridge, Cambridge CB2 3EB, UK; Wolfson Brain Imaging Centre, Department of Clinical Neurosciences, University of Cambridge, Cambridge CB2 0QQ, UK; Department of Neurology, University of Maryland School of Medicine, Baltimore, MD 21201, USA; Wellcome Sanger Institute, Wellcome Genome Campus, Hinxton, Cambridge CB10 1SA, UK; Spectrum Health, Helen DeVos Children’s Hospital, Grand Rapids, MI 49503, USA; Wellcome Sanger Institute, Wellcome Genome Campus, Hinxton, Cambridge CB10 1SA, UK; Wellcome Sanger Institute, Wellcome Genome Campus, Hinxton, Cambridge CB10 1SA, UK; Department of Clinical Genetics, Alder Hey Children’s Hospital, Liverpool L14 5AB, UK; Department of Genetics, University Medical Centre, University of Groningen, Groningen 9713 GZ, The Netherlands; Wellcome Sanger Institute, Wellcome Genome Campus, Hinxton, Cambridge CB10 1SA, UK; Haven Clinical Psychology Practice Ltd, Bude, Cornwall EX23 9HP, UK; IGBMC, UMR7104, INSERM, Illkirch 67404, France; Department of Clinical Genetics, Erasmus MC, University Medical Center Rotterdam, Rotterdam 3015 GD, The Netherlands; Department of Clinical Genetics, Leiden University Medical Center, Leiden 2300 RC, The Netherlands; Wellcome Sanger Institute, Wellcome Genome Campus, Hinxton, Cambridge CB10 1SA, UK; Open Targets, Wellcome Genome Campus, Hinxton, Cambridge CB10 1SA, UK; Department of Genetics, University of Cambridge, Cambridge CB2 3EH, UK; Laboratory for Diagnostic Genome Analysis, Department of Clinical Genetics, Leiden University Medical Center, Leiden 3015 GD, The Netherlands; Wellcome Sanger Institute, Wellcome Genome Campus, Hinxton, Cambridge CB10 1SA, UK; Institute of Metabolic Science, Cambridge University, Cambridge CB2 0QQ, UK; Wellcome Sanger Institute, Wellcome Genome Campus, Hinxton, Cambridge CB10 1SA, UK; Faculty of Health and Medical Science, University of Surrey, Guildford GU2 7YH, UK; Wellcome Sanger Institute, Wellcome Genome Campus, Hinxton, Cambridge CB10 1SA, UK; New Leaf Center, Clinic for Special Children, Mount Eaton, OH 44659, USA; Oxford Centre for Genomic Medicine, Oxford University Hospitals NHS Trust, Oxford OX3 7HE, UK; London Health Sciences Centre, London, ON N6A 5W9, Canada; Division of Medical Genetics, Department of Pediatrics, Schulich School of Medicine and Dentistry, Western University, London, ON N6A 5W9, Canada; Department of Neurology, Children’s National Medical Center, Washington DC 20007, USA; Wellcome Sanger Institute, Wellcome Genome Campus, Hinxton, Cambridge CB10 1SA, UK; Haven Clinical Psychology Practice Ltd, Bude, Cornwall EX23 9HP, UK; Department of Clinical Genetics, Aarhus University Hospital, Aarhus DK-8200, Denmark; Department of Genetics, University Medical Centre, University of Groningen, Groningen 9713 GZ, The Netherlands; Department of Clinical Genetics, University Hospitals of Leicester, Leicester Royal Infirmary, Leicester LE1 7RH, UK; New Leaf Center, Clinic for Special Children, Mount Eaton, OH 44659, USA; Institute of Human Genetics, International Centre for Life, Newcastle upon Tyne NE1 7RU, UK; Wellcome Sanger Institute, Wellcome Genome Campus, Hinxton, Cambridge CB10 1SA, UK; Qkine Ltd., Cambridge CB5 8HW, UK; Wellcome Sanger Institute, Wellcome Genome Campus, Hinxton, Cambridge CB10 1SA, UK; Wellcome Sanger Institute, Wellcome Genome Campus, Hinxton, Cambridge CB10 1SA, UK; Wellcome Sanger Institute, Wellcome Genome Campus, Hinxton, Cambridge CB10 1SA, UK; Institute of Metabolic Science, Cambridge University, Cambridge CB2 0QQ, UK; Department of Neurology, University of Maryland School of Medicine, Baltimore, MD 21201, USA; INSERM Unit 1231, Université de Bourgogne Franche-Comté, Dijon 21078, France; RILD Wellcome Wolfson Medical Research Centre, University of Exeter, Exeter EX2 5DW, UK; RILD Wellcome Wolfson Medical Research Centre, University of Exeter, Exeter EX2 5DW, UK; Peninsula Clinical Genetics Service, Royal Devon University Healthcare NHS Foundation Trust, Exeter EX1 2ED, UK; Wellcome Sanger Institute, Wellcome Genome Campus, Hinxton, Cambridge CB10 1SA, UK; Waltham Petcare Science Institute, Waltham on the Wolds LE14 4RT, UK; Wellcome Sanger Institute, Wellcome Genome Campus, Hinxton, Cambridge CB10 1SA, UK; Open Targets, Wellcome Genome Campus, Hinxton, Cambridge CB10 1SA, UK; Wellcome Sanger Institute, Wellcome Genome Campus, Hinxton, Cambridge CB10 1SA, UK; Open Targets, Wellcome Genome Campus, Hinxton, Cambridge CB10 1SA, UK

**Keywords:** mTOR, neurodevelopmental disorders, recessive, animal model, iPSC, macrocephaly

## Abstract

*KPTN*-related disorder is an autosomal recessive disorder associated with germline variants in *KPTN* (previously known as kaptin), a component of the mTOR regulatory complex KICSTOR. To gain further insights into the pathogenesis of *KPTN*-related disorder, we analysed mouse knockout and human stem cell KPTN loss-of-function models.

*Kptn* ^−/−^ mice display many of the key *KPTN*-related disorder phenotypes, including brain overgrowth, behavioural abnormalities, and cognitive deficits. By assessment of affected individuals, we have identified widespread cognitive deficits (*n* = 6) and postnatal onset of brain overgrowth (*n* = 19). By analysing head size data from their parents (*n* = 24), we have identified a previously unrecognized KPTN dosage-sensitivity, resulting in increased head circumference in heterozygous carriers of pathogenic *KPTN* variants.

Molecular and structural analysis of *Kptn*^−/−^ mice revealed pathological changes, including differences in brain size, shape and cell numbers primarily due to abnormal postnatal brain development. Both the mouse and differentiated induced pluripotent stem cell models of the disorder display transcriptional and biochemical evidence for altered mTOR pathway signalling, supporting the role of KPTN in regulating mTORC1.

By treatment in our *KPTN* mouse model, we found that the increased mTOR signalling downstream of KPTN is rapamycin sensitive, highlighting possible therapeutic avenues with currently available mTOR inhibitors. These findings place *KPTN*-related disorder in the broader group of mTORC1-related disorders affecting brain structure, cognitive function and network integrity.

## Teaser

Levitin *et al.* model *KPTN*-related disorder in knockout mice and in human cells. Consistent with findings in patients, the mouse model shows cognitive and behavioural changes, as well as significant brain overgrowth. Reversible mTOR pathway disruption is evident in both models, suggesting therapeutic potential of mTOR inhibitors.

## Introduction

Human brain development requires an intricate and tightly regulated sequence of events involving cell proliferation, migration, differentiation and integration into cohesive circuitry,^1^ complex processes that are highly sensitive to changes in gene expression. Neurodevelopmental disorders (NDDs) with underlying genetic causes have been associated with most stages of brain development.^2^ Prevalent phenotypic manifestations include changes in brain volume and cranial anomalies.^3-5^ A minority of these size changes involve head overgrowth (e.g. 1250 out of 5255, or 24%, of cases in the Decipher open access patient collection^6^ where a head size change was observed), termed ‘macrocephaly’ where the occipitofrontal circumference (OFC) exceeds two standard deviations (SD). Megalencephaly is typically used to describe such overgrowth when it specifically affects brain structures (neurons, glia, ventricles), rather than simply the anatomical dimensions of the head and skull.^7,8^

For overgrowth disorders, there is a frequent convergence on a smaller set of growth regulating pathways, including AKT and mTOR (mechanistic target of rapamycin) signalling.^9^ mTOR, a serine/threonine kinase that forms the catalytic subunit of two distinct protein complexes known as mTOR complex 1 (mTORC1) and 2 (mTORC2),^10-13^ regulates protein synthesis, cell growth and nutrient uptake. Moreover, mTOR signalling is central to the regulation of long-lasting synaptic plasticity, which in turn is critical to the formation and persistence of memories.^14^ Aberrant mTOR signalling is associated with numerous diseases such as cancer, diabetes and neurological disorders including epilepsy, autism, developmental delay and macrocephaly.^10-13^*De novo* and inherited variants have been identified in numerous mTOR pathway genes, leading to the term ‘mTORopathies’ to describe the involvement of the mTORC1 signalling pathway in these disorders.^15-19^ A growing body of literature is uncovering the intricate relationship between the mTOR pathway, neural stem cell proliferation and neuronal differentiation,^20-23^ providing insights into the mechanisms of malformations of cortical development such as tuberous sclerosis complex, focal cortical dysplasia, hemimegalencephaly and megalencephaly.^24-26^

We previously described inherited biallelic variants in the *KPTN* gene as underlying KPTN-related disorder (KRD), also known as macrocephaly, autistic features, seizures, developmental delay (MASD) syndrome (OMIM 615637), an autosomal recessive neurodevelopmental disorder in nine affected individuals from an extended interrelated family of Ohio Amish background.^27^ All affected individuals were either homozygous or compound heterozygous for two *KPTN* founder variants [(p.(Ser259*) and p.(Met241_Gln246dup)]. The cardinal clinical features of KRD include craniofacial dysmorphism (frontal bossing, long face with prominent chin, broad nasal tip and hooded eyelids), macrocephaly with OFC measurements up to 5.4 SD above the mean, hypotonia in infancy, global developmental delay, intellectual disability (mild to severe), seizures and behavioural features including anxiety, hyperactivity, stereotypies and repetitive speech. Where available, neuroimaging revealed a globally enlarged but structurally normal brain. Subsequent studies have identified six additional affected non-Amish individuals with biallelic *KPTN* variants and a similar clinical presentation. To date, five distinct variants in *KPTN* have been published^28-31^ that were identified in KRD probands.

KPTN has been identified as a negative upstream regulator of the mTORC1 signalling pathway as part of the KICSTOR protein complex that also includes SZT2, ITFG2 and C12orf66.^32^ In cell lines, KICSTOR is required for the inhibition of mTORC1 signalling in response to amino acid or glucose deprivation.^11,32^ KPTN forms a heterodimer with ITFG2, which then complexes with SZT2. Interestingly, biallelic variants in *SZT2* also cause a distinct NDD, characterized by severe early-onset epileptic encephalopathy, global developmental delay, structural brain abnormalities and frequent macrocephaly.^16,17^ In mice, *Szt2* loss-of-function (LoF) mutants display seizure susceptibility and increased mTORC1 signalling in the brain.^15,33^

We aimed to assess the functional consequences of homozygous *Kptn* LoF in mice [referred to here as *Kptn* knockout (KO) mice], as well as human induced pluripotent stem cell (iPSC) models of the condition, as to date no study has modelled KRD *in vivo* or *in vitro*. Our mouse model recapitulates the primary human phenotypes, including cognitive and behavioural impairments and skull and brain overgrowth. Molecular findings include the misexpression of numerous seizure-associated genes, progenitor markers, as well as biochemical and transcriptional evidence for increased mTOR signalling. The aberrant mTOR signalling is corrected by acute treatment with rapamycin, a known mTOR inhibitor, supporting a preclinical path to therapeutic intervention. Cortical neural precursors differentiated from human iPSCs null for *KPTN* robustly support the mouse findings. We also identify a previously unrecognized effect of head overgrowth in heterozygous carriers of KPTN alleles, consistent with the recent studies showing that heterozygosity of alleles for other recessive disorders may be contributing to incompletely penetrant phenotypes in the general population.^34-37^ Together, these models confirm *KPTN* to be among a growing list of mTOR modulating disease-associated genes and provide a platform for further exploring the pathomechanistic basis of KRD specifically, and macrocephaly more generally.

## Materials and methods

### Mouse production

The *Kptn^tm1a(EUCOMM)Wtsi^* mouse model was generated at the Wellcome Sanger Institute, and kept on a C57BL/6NTac background (Taconic). All mouse production and testing were performed according to UK government and institutional policies as detailed below (Study approval). Further details are described in the Supplementary material, ‘Methods’ section.

### Mouse behavioural paradigms

In all assays, the mice were tracked using infrared video cameras and automated video tracking software Ethovision XT 8.5 (Noldus). All experiments were carried out on mice aged 10–22 weeks. Mice were handled for 2–3 days before testing, and habituated to the testing room in their home cages for ≥30 min under the same light condition as the test. Male mice were tested unless stated otherwise. Full details of the open field, light/dark box, social recognition, pairwise discrimination and Barnes maze testing are described in the Supplementary material, ‘Methods’ section.

### Transcardial perfusions

Mice were terminally anaesthetized by intraperitoneal injection of 0.1 ml pentobarbitone sodium. After the mice were no longer responsive, 20 ml of cold PBS was injected into the left ventricle of the exposed heart at low speed, followed by 20 ml of 4% paraformaldehyde (PFA), either by hand or using a perfusion pump. After the perfusion, skulls and brain segments were collected.

### MRI

For MRI analysis, skulls were collected after transcardial perfusion and stored in formalin solution at 4°C. MRI and analysis were performed, using previously published methodology.^38^ For female mice, we used *n* = 8 for each genotype, For male mice, we used six wild-type and seven homozygous mutant animals. Full details are described in the Supplementary material, ‘Methods’ section.

### Microcomputed X-ray tomography

Skulls of male mice (*n* = 5 per genotype) were collected after transcardial perfusion and stored in formalin solution at 4°C until they were ready to be scanned. High resolution images were acquired using the 1172 Bruker microCT scanner. Inter-landmark distances were calculated, and significant differences in mean linear distances were identified using a Student's *t*-test (α = 0.05). Full details are described in the Supplementary material, ‘Methods’ section.

### Histomorphometric analysis and immunohistochemistry

The histomorphological analysis was carried out on adult mice aged 16 weeks (*n* = 8 per genotype and sex), as previously described.^39,40^ Mice were separately analysed according to sex. Immunohistochemistry for mTOR signalling was performed on paraffin sectioned material from adult mice aged 16 weeks. Full details are described in the Supplementary material, ‘Methods’ section.

### Tissue lysis and immunoblotting

For protein extracts used in immunoblotting, PBS perfused tissue was collected as described above, snap frozen in liquid nitrogen, and stored in −80°C. Full details are described in the Supplementary material, ‘Methods’ section.

### Rapamycin treatment

We freshly dissolved stock rapamycin (dissolved in ethanol 20 mg/ml) (LC Laboratories) in vehicle solution (5% Tween, 5% PEG, ethanol, in distilled water) before use. We administered rapamycin intraperitoneally once daily at a dose of 5 mg/kg for 3 days before and on the day of collecting tissue for immunoblotting.

### Mutagenesis and differentiation of KPTN iPSC models

IPSC experiments were all performed in an isogenic background: Kolf2C1_WT (HPSI0114i-kolf_2), feeder-free hiPSC, male, derived from skin tissue using Cytotune 1 reprogramming.^41^ To create knockout iPSC lines, a guide RNA (gRNA, GRCh38:19:47479882-47479904) was selected to target coding exon 8 of the human *KPTN* gene. Further details of gRNA delivery, mutagenesis and clonal isolation are described in the Supplementary material, ‘Methods’ section. This work was performed by the Gene Editing facility at the Wellcome Sanger Institute.

### Differentiation of iPSCs to cortical neural precursor cells

*KPTN* mutant heterozygous, homozygous edited and wild-type control iPSC lines were cultured in feeder-free conditions as per published protocols.^42,43^ Dual-SMAD inhibition (SB431542, #ab120163, Abcam and LDN193189, #2092-5, Cambridge Bioscience) was used in a 10-day neural induction in the presence of XAV939 (#3748, Tocris) to promote regional forebrain identity.^77,108^ All wild-type, heterozygous and homozygous clones (*n* = 2, 2, 1, respectively) were differentiated in duplicate, providing two to four separate biological replicates per genotype (*n* = 4, 4, 2 replicates, respectively). Complete details can be found in Supplementary material, ‘Methods’ section.

### IPSC RNA extraction and RNA sequencing

RNA extraction and sequencing of iPSC-derived materials was performed using manufacturer's protocols for the RNeasy QIAcube kit on a QIAcube automated system (Qiagen). RNA sequencing libraries were generated, sequenced and analysed as described in the Supplementary material, ‘Methods’ section. Briefly, the data preprocessing was done with a custom Nextflow pipeline (https://github.com/wtsi-hgi/nextflow-pipelines/blob/rna_seq_5607/pipelines/rna_seq.nf), which includes the aligner parameters. Differential gene expression was analysed using the DESeq2 package^44^ with SVA correction.^45^ An adjusted *P*-value threshold of 0.05 was selected to identify significant differences between wild-type and mutant samples.

### Mouse RNA extraction and RNA sequencing

For wild-type and homozygous *Kptn* mouse samples, we used *n* = 5–6 per genotype and tissue as follows (wild-type/mutant): E18 brain, *n* = 6/6; P21 hippocampus, *n* = 5/6; P21 cortex, *n* = 5/6; adult cerebellum, *n* = 5/6; adult hippocampus, *n* = 6/6; adult cortex, *n* = 5/5. Full details of extraction are described in the Supplementary material, ‘Methods’ section. Library preparation was performed by Wellcome Sanger Institute DNA Pipelines, as described above for iPSCs. Samples were sequenced using 75 bp paired-end sequencing reads on a Illumina-HTP HiSeq 4000 system. Downstream processing and analysis are detailed in the Supplementary material, ‘Methods’ section. Briefly, the data preprocessing was done with a Nextflow pipeline (https://github.com/wtsi-hgi/nextflow-pipelines/blob/rna_seq_mouse/pipelines/rna_seq.nf). The count data were used as input for differential gene expression analysis using DESeq2 package^44^ with SVA correction.^45^ The default DESeq2 cut-off of BH-adjusted *P*-value <0.1 was used for the mouse RNA-Seq analyses.

For the identification of functionally enriched terms in the differentially expressed genes, Gene ontology (GO) and KEGG pathway enrichment analyses was performed using the grpofiler online suite^46^ (http://biit.cs.ut.ee/gprofiler/index.cgi). A threshold of 5% false discovery rate (FDR) and an enrichment significance threshold of *P* < 0.05 (hypergeometric test with Benjamini-Hochberg FDR correction for multiple testing) was used. In all analyses, a background comprised of only the expressed genes in the tissue studied [genes where the adjusted *P*-value yielded a numerical value, different to NA (not available)].

### Clinical and genetic studies

Affected individuals were identified by their clinician, using GeneMatcher^47^ and through the published medical literature. Research was performed with informed consent from the study participants or their legal guardians, as detailed below. Affected individuals and family members were investigated according to routine clinical standards for the diagnosis of developmental delay/intellectual disability and neurological disease. Genetic studies were performed as part of clinical and/or research investigations dependent on clinical presentation and family history. Detailed phenotype and genotype information was obtained by the clinical care provider using a targeted questionnaire to identify specific clinical features including OFC. All OFC *z*-scores were recalculated using measurements and known age at assessment using the British 1990 growth reference^48^ to ensure all values were comparable. *Z*-score conversions around birth are gestation-adjusted where needed.

### Psychometric testing of *KPTN*-related disorder probands

Six Amish individuals between the ages of 11 and 29 with KRD (three female, three male) were psychometrically assessed using the Wechsler Intelligence Scale for Children 4th Edition (WISC-IV) (6–16 years) or the Wechsler Adult Intelligence Scale 4th Edition (WAIS-IV) (over 16 years), which assesses cognitive performance in four domains including verbal comprehension (VCI), perceptual reasoning (PRI), processing speed (PSI) and working memory (WMI) indices, and can be combined to generate a full-scale intelligence quotient (FSIQ) score. Further details are provided in the Supplementary material, ‘Methods’ section.

### CMAP query

To identify anti-correlated transcriptional profiles in the CMAP database,^49^ we selected the 150 most strongly up- and downregulated genes from our differential expression analysis of *KPTN*^−/−^ neural precursor cells (NPCs), and queried the CMAP via their online tool (https://clue.io/query) using the L1000 TOUCHSTONE (V1.1.1.2) gene expression dataset.

### Statistics

All statistical tests are listed in the relevant sections, and in Supplementary material, ‘Methods’ section.

### Study approval

All mouse work was carried out under UK Home Office licenses, in accordance with the Animal Welfare and Ethical Review Body of the Wellcome Sanger Institute and the UK Home Office Animals (Scientific Procedures) Act of 1986, under UK Home Office PPL 80/2472 and PPL P6320B89B. Human patient research was performed with informed consent from the study participants or their legal guardians, according to institutional and international guidelines for studies with human participants and materials. Institutional study approvals include: Akron Children's Hospital (IRB 986876-3), University of Arizona (IRB 10-0050-010), University of Exeter Medical School.

## Results

### Generation and characterization of a KPTN mouse model

The majority of variants associated with KRD result in premature termination and likely inactivate KPTN protein function,^30^ by introducing either a frameshift or a novel nonsense codon.^30^ Therefore, in order to reproduce the type of allele seen in KRD, we used an engineered LoF mouse model.^50^ The allele introduces a splice-trapping cassette into an intron downstream of coding exon 8, resulting in an in-frame LACZ open reading frame and premature termination of transcription via an exogenous polyadenylation signal. The resulting transcript closely resembles the truncating allele [p.(Ser259*)] observed in the original KRD publication^27^ (Supplementary Fig. 1A), which also led to premature termination in exon 8. Homozygous *Kptn* KO mice are observed at expected near-Mendelian ratios and appear to have normal body shape. Read count quantification in bulk RNA-Seq shows a robust reduction in *Kptn* mRNA pre- and postnatally, and across adult brain regions (87–90% decrease, Supplementary Fig. 1B), in the homozygous *Kptn* KO mice. This confirms that the mouse model is functioning efficiently as a LoF allele and leads to nonsense-mediated decay of *Kptn* mRNA *in vivo*.

### *Kptn* ^−/−^ mice have increased locomotor activity and anxiety-like phenotypes

**Figure 1:**
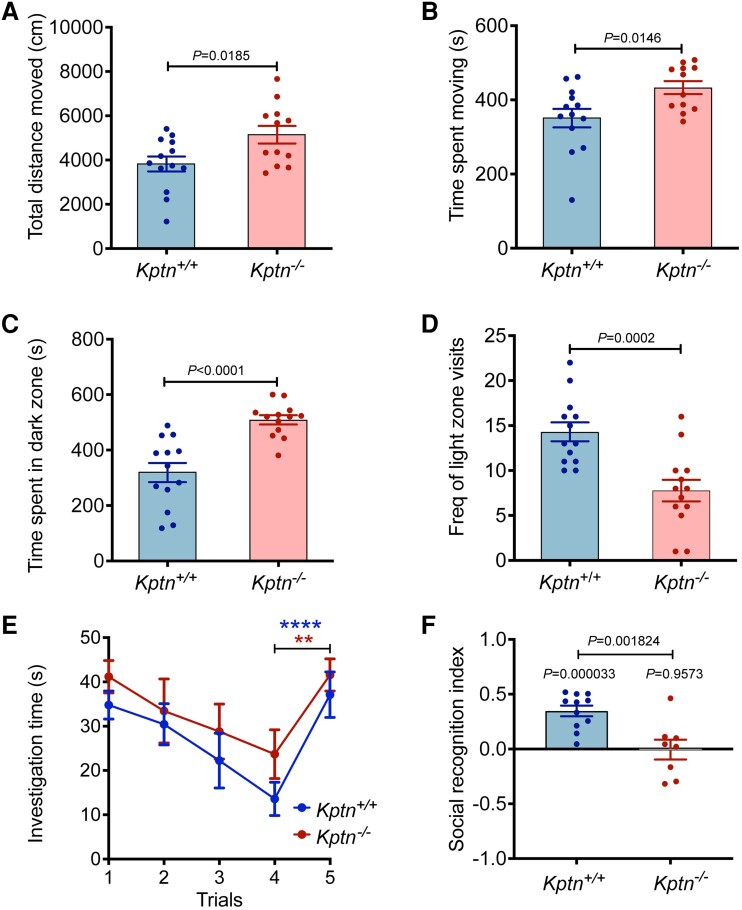
***Assessing Kptn* ^−/−^ mice for changes in activity, anxiety and memory**. *Kptn*^−/−^ mice were tested for locomotor activity (**A** and **B**) altered anxiety-like behaviour (**C** and **D**) and memory retention (**E** and **F**). (**A** and **B**) Changes in behaviour in the open field assay of *Kptn*^−/−^ mice (*n* = 12) compared to wild-type controls (*Kptn*^+/+^, *n* = 13). (**A**) Distance covered (*P* = 0.0185, *t* = 2.534 df = 23, two-tailed Student’s *t*-test); (**B**) time spent moving (*P* = 0.0146, *t* = 2.64 df = 23, two-tailed Student’s *t-*test). (**C** and **D**) Results of anxiety testing using a light/dark box, comparing *Kptn*^−/−^ mice (*n* = 13) and wild-type controls (*Kptn*^+/+^, *n* = 13). (**C**) Time spent in the dark zone (*P* < 0.0001; *t* = 4.946 df = 24, two-tailed Student’s *t*-test) and (**D**) frequency of visits to light zone (*P* = 0.0002, *t* = 4.326 df = 25, two-tailed Student’s *t*-test). (**E**) Memory testing using social recognition assay, measuring investigation time on Day 1 by wild-type controls (*Kptn*^+/+^*n* = 11) and *Kptn* mutant mice (*Kptn*^−/−^, *n* = 8) of a stimulus animal repeatedly presented to them over the course of four trials. To assess the ability to discriminate novel from familiar mice, in Trial 5 all animals are presented with a novel stimulus animal (change in investigation Trials 1–4 versus Trial 5, two-way ANOVA, interaction Trial × Genotype *F*(4,68) = 0.3852, *P* = 0.8185; Trial *F*(4,68) = 16.26, *P* < 0.0001; *post hoc* analysis of Trial 4 versus Trial 5, *Kptn*^+/+^*P* < 0.0001****, *Kptn*^−/−^*P* = 0.0023**). (**F**) Assessment of 24 h social recognition memory using the social recognition index, which indicates the difference in investigation of unfamiliar stimulus versus the familiar stimulus mouse (from Day 1) as a fraction of total investigation time (see ‘Materials and methods’ section) for wild-type *Kptn*^+/+^ controls (*n* = 11), and *Kptn*^−/−^ mice (*n* = 8) (between genotypes, two-tailed Student’s *t*-test; within genotypes, one sample two-tailed Student’s *t*-tests against mean of zero).Values are plotted as mean ± standard error of the mean (SEM).

The majority of individuals with KRD display consistent neurobehavioural traits, including intellectual impairment and features suggestive of autistic spectrum disorder (anxiety, stereotypies and repetitive speech) with variable hyperactivity and seizures.^27-31^ To investigate whether our mouse model recapitulates any of the neurobehavioural features of the human disorder, we evaluated *Kptn* KO mice with a series of functional tests. Locomotor capabilities, including distance covered and time spent moving, were assessed in *Kptn*^−/−^ mice compared to wild-type controls, using video-assisted observation and scoring in an open-field test.^51^*Kptn*^−/−^ mice travelled a significantly greater distance than *Kptn*^+/+^ controls, accounted for by the increase in time spent moving (*P* < 0.05) (Fig. 1A and B). All other activity parameters were not significantly different between genotypes (data not shown). Anxiety-related behaviours are a phenotype observed in >50% of individuals with KRD.^30^ To assess anxiety in *Kptn*^−/−^ mice, we performed a light/dark box test.^52,53^*Kptn*^−/−^ mice spent significantly more time in the dark zone (59.6% increase, *P* = 4.8 × 10^−5^), and had reduced frequency of visits to the light zone (45.6% reduction, *P* = 0.0006) (Fig. 1C and D, respectively). These results indicate hyperactivity and a strong anxiety-like phenotype in the *Kptn*^−/−^ mouse model, concordant with those observed in KRD.^27-31^

### *Kptn* ^−/−^ mice have cognitive deficits

KRD in humans is characterized by highly penetrant intellectual disability. Thus, we evaluated whether *Kptn*^−/−^ mice display concordant intellectual deficits. First, an ethologically relevant social recognition (SOR) assay was used to assesses olfactory-mediated hippocampus-dependent memory.^54-56^ On Day 1, *Kptn*^−/−^ mice display no detectable deficit in social interaction, as measured by the time spent investigating the novel conspecific (Stimulus A) on Trial 1 (Fig. 1E). With repeated exposure over four trials, both wild-type and *Kptn*^−/−^ mice habituated to the stimulus animal, indicating a functional olfactory system. Upon presentation of a novel stimulus animal, both genotypes significantly increased their investigation time (Trial 5), indicating successful recognition that this stimulus was novel (Fig. 1E). On Day 2, when given the choice between investigating a new, unfamiliar mouse and a familiar one (Stimulus A from Day 1), wild-type mice spent ∼40% more time investigating the unfamiliar mouse (Fig. 1F). *Kptn*^−/−^ mice, however, have a significantly reduced social recognition index compared to wild-types (mean of −0.005 in *Kptn*^−/−^ mice versus 0.347 in wild-types, *P* = 0.001824, two-sample two-tailed Student's *t*-test), and did not show a preference for the unfamiliar mouse (Fig. 1F), indicating a reduced recollection of the previous exposure. The results of the SOR assay indicate a memory deficit in *Kptn*^−/−^ mice.

**Figure 2:**
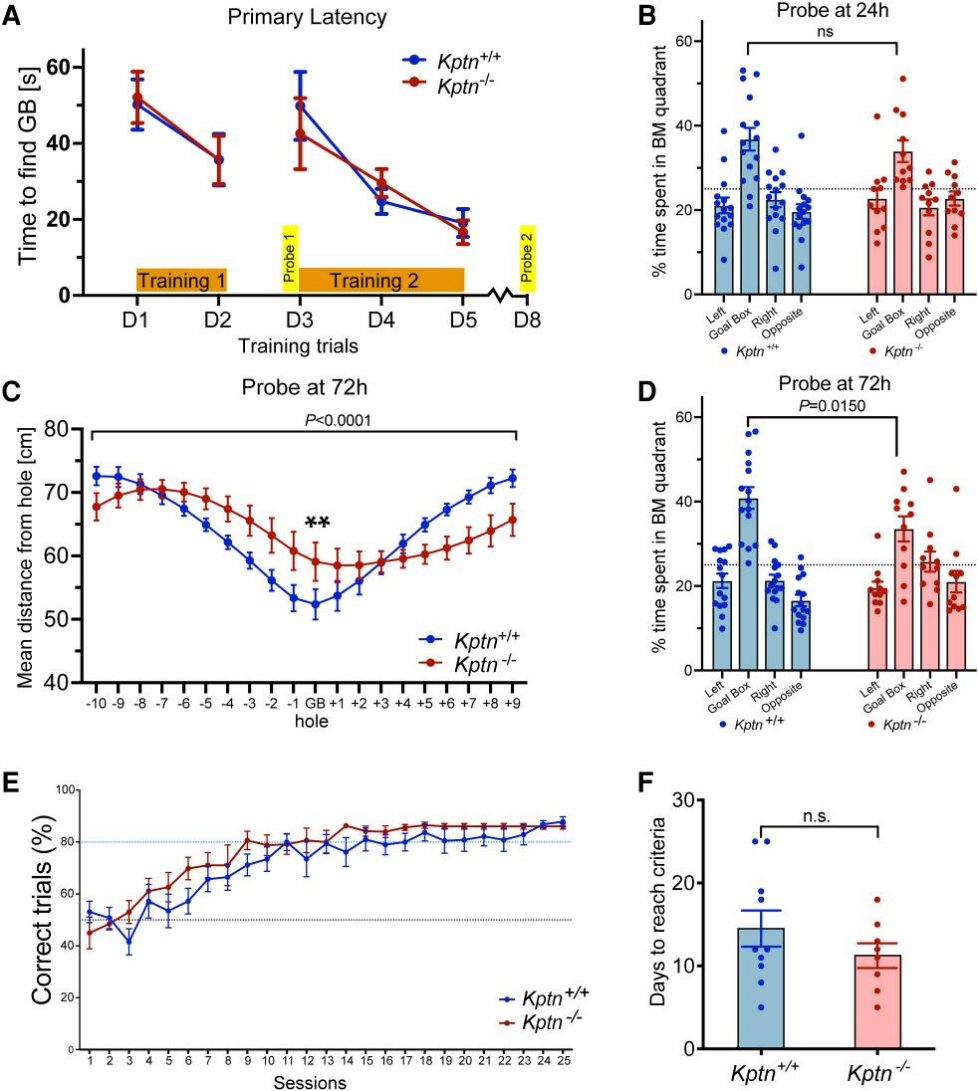
**Performance of *Kptn*^−/−^ mice in memory assays.** (**A**–**D**) Barnes maze applied to KRD mouse model to assess spatial memory. (**A**) Time taken to find the escape box (primary latency) across all days of training (Training 1, D1–D2; Training 2, D3–D5), comparing *Kptn*^+/+^ (*n* = 15) and *Kptn*^−/−^ (*n* = 11) mice [two-way ANOVA, interaction Genotype × Zone *F*(4,120) = 0.2577, *P* = 0.9044]. (**B**) The percentage of time *Kptn*^+/+^ (blue, *n* = 15) and *Kptn*^−/−^ (red, *n* = 11) mice spent around the target hole quadrant (Goal Box) and non-target quadrants of the Barnes maze during a probe trial 24 h after Training 1 [two-way ANOVA, interaction Genotype × Zone *F*(3,96) = 0.8544, *P* = 0.4676]. Both genotypes spent significantly more time near the target versus all other holes (*post hoc* FDR q < 0.0003. *P* ≤ 0.001). (**C**) The mean distance from each hole during probe trial, 72 h after Training 2, comparing *Kptn*^−/−^ and *Kptn*^+/+^ mice [two-way ANOVA, interaction Genotype × Hole *F*(19,500) = 5.351, *P* = <0.0001; *post hoc* on GB, FDR q = 0.0045, *P* = 0.0014**]. (**D**) The percentage of time *Kptn*^+/+^ (blue, *n* = 15) and *Kptn*^−/−^ (red, *n* = 11) mice spent in the target hole quadrant (Goal Box) and non-target quadrants of the Barnes maze during the probe trial, 72 h after Training 2, comparing time in the target hole quadrant between *Kptn*^−/−^ and *Kptn*^+/+^ mice [two-way ANOVA, interaction Genotype × Zone *F*(3,96) = 3.667, *P* = 0.0150; *post hoc* FDR q = 0.0632, *P* = 0.0150] (**E** and **F**) Assessment of *Kptn^−/−^* mice in hippocampus-independent pairwise discrimination task (Bussey-Saksida chamber). (**E**) Percentage of correct trials (when CS+ image was nose-poked) out of the total trials completed per session for *Kptn*^−/−^ (*n* = 8) and *Kptn*^+/+^ (*n* = 10) mice [two-way ANOVA, interaction Genotype × Session *F*(1,16) = 1.144, *P* = 0.3007]. (**F**) Number of days to reach criteria comparing *Kptn*^−/−^ and *Kptn*^+/+^ mice (*P* = 0.2093, *t* = 1.162 df = 16, two-tailed Student's *t*-test). Values are plotted as mean ± standard error of the mean (SEM).

To further characterize the memory deficit, *Kptn*^−/−^ mice were tested in a spatial memory assay, the Barnes maze,^57-59^ which is similar in principle to the Morris water maze and tests a mouse's ability to identify an escape box using spatial cues in an open arena. During both training periods (Training 1 and 2), there was no difference between *Kptn*^−/−^ and wild-type mice in the time taken to approach the escape box (primary latency to approach), indicating that both genotypes were able to locate the target zone with equivalent speed (Fig. 2A). During the first probe test, after 24 h (when the escape box was removed), the time spent around the target hole relative to other holes was measured (Fig. 2B). Both genotypes spent longer around the target hole quadrant when compared to other quadrants (*P* ≤ 0.05). However, during the second probe test (72 h after the end of Training 2), the *Kptn*^−/−^ mice do show a deficit in long-term memory, and were on average further from the goal box location (Fig. 2C, 12.8% further, *P* = 0.0014), and spent significantly less time in the target zone than wild-type controls (Fig. 2D, 17.9% less time, *P* = 0.0150).

The memory impairments identified in *Kptn*^−/−^ mice with the SOR and Barnes maze assays indicate a likely deficit in hippocampus-dependent memory retention. To test whether the *Kptn* KO mice also display impairments in tasks less reliant on hippocampal function, we used pairwise discrimination, a visual operant conditioning task using a touchscreen platform.^60-63^ During the pairwise discrimination task, both genotypes selected the correct image ∼50% of the time in Session 1 (Fig. 2E, *Kptn*^−/−^, 45%; *Kptn^+/+^*, 53%), indicating that the mice had no innate bias for either of the images at the start of the task. The mean number of days to reach criteria (80% correct trials for two consecutive days^61^) was not significantly different between genotypes, and *Kptn*^−/−^ mice performed no worse than *Kptn*^+/+^ mice (*Kptn*^−/−^, 11.25 days; *Kptn*^+/+^, 14.5 days) (Fig. 2F). We performed a single reversal trial to confirm that the mice were capable of discriminating between images. As expected, the percentage of correct trials dropped significantly below 50% for both genotypes when the CS+ (rewarded conditioned stimulus) and CS− (non-rewarded conditioned stimulus) images were swapped (*Kptn*^−/−^, 24%; *Kptn*^+/+^, 19.2%), indicating a strong association with the original CS+ image (data not shown). These data indicate that *Kptn*^−/−^ mice perform at least as well as wild-type animals in the pairwise discrimination task. The results of our three independent memory tests suggest that *Kptn* KO mice experience selective deficits, with at least some memory sparing.

### Widespread cognitive deficits in individuals with *KPTN*-related disorder

**Figure 3:**
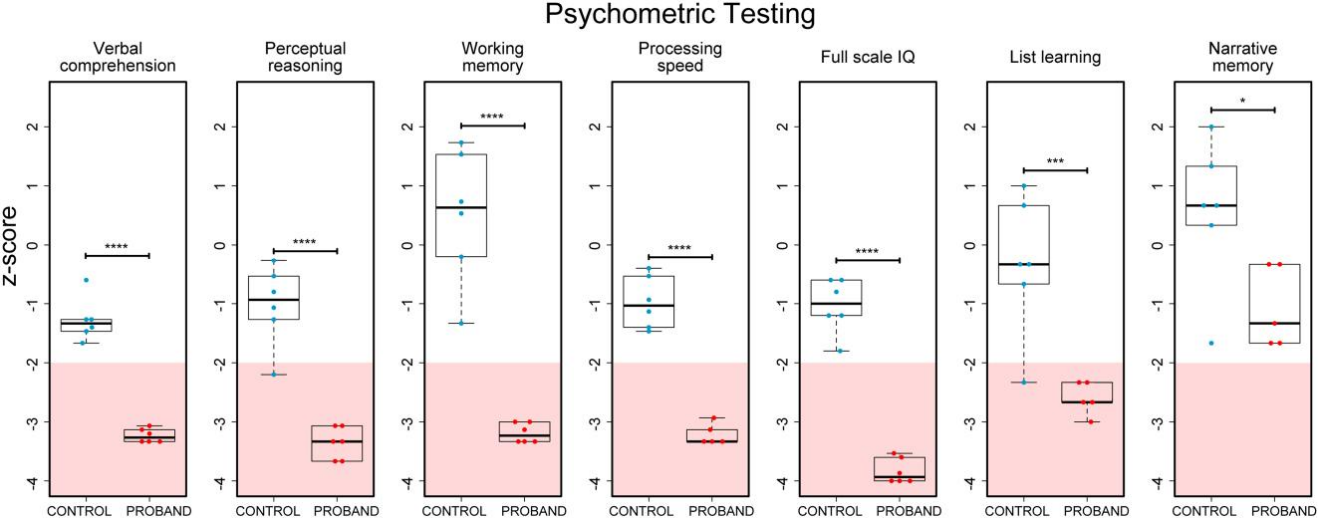
**Psychometric testing of Amish individuals with KRD reveals widespread cognitive deficits**. Six Amish individuals between the ages of 11 and 29 with KRD (three female, three male) and age-matched population controls were psychometrically assessed (see ‘Materials and methods’ section) to measure cognitive performance in four domains including verbal comprehension, perceptual reasoning, processing speed and working memory indices, that can be combined to generate a full-scale intelligence quotient score. Auditory-verbal recall performance was also assessed by list learning and story memory tests. The impaired range is indicated by pink shading, *z-*score <2. Scores from KRD individuals were found to be significantly lower than control individuals in all tests administered (*P* < 0.05, two-tailed Student's *t*-tests). *P < 0.05; ***P < 0.001; ****P < 0.0001.

To gain further insights into the range of cognitive traits of patients with KRD, we identified six Amish individuals with KRD (aged 11–29, three female and three male individuals) (Supplementary Fig. 2) and six age-matched Amish control individuals for psychometric testing. All 12 were assessed VCI, PRI, PSI, WMI, FSIQ, list learning and narrative memory. We detected significant impairment of cognitive function in all individuals with KRD, with the majority of scores in the impaired range (*z*-score <−2). The degree of impairment in KRD on narrative memory shown in our study was milder, however the difference between KRD and control individuals was significant (*P* = 0.02886, Fig. 3).^64^ These psychometric test findings, revealing widespread cognitive deficits, highlight the concordance of cognitive phenotypes between our mouse model and the human disorder.

### *Kptn* deficiency is associated with severe and progressive macrocephaly in mice

Macrocephaly and frontal bossing are frequently reported features of KRD in humans. MRI neuroimaging typically reveals isolated generalized megalencephaly. We therefore investigated whether the *Kptn*^−/−^ mice also replicate this phenotype. Microcomputed tomography (μCT), MRI and histological morphometrics were used to quantify changes in skull and brain volume, shape and cellular distribution.^64^

**Figure 4:**
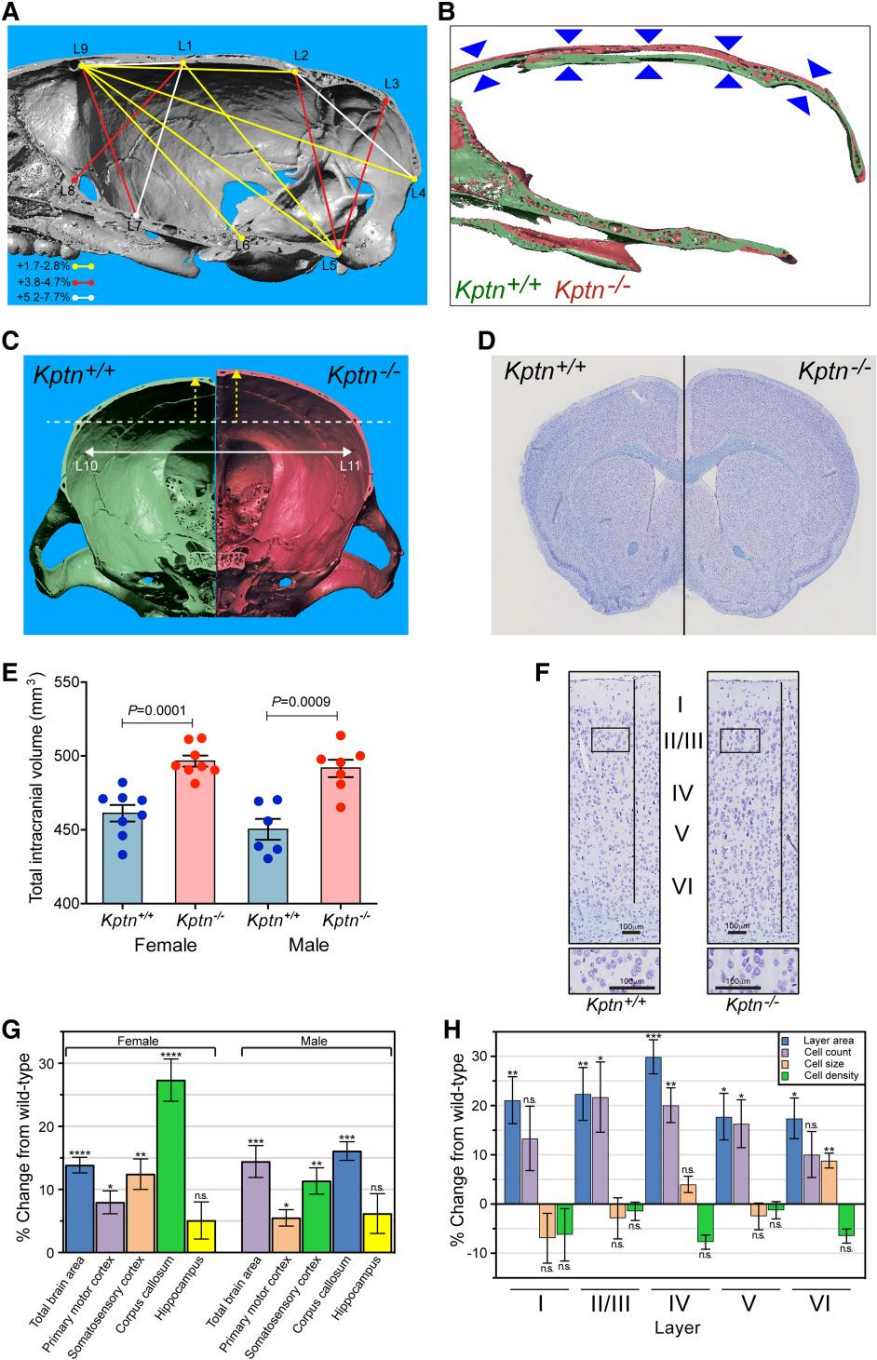
**Assessing *Kptn*^−/−^ mouse model skull and brain morphology**. Micro-computed X-ray tomography reconstructions were collected and analysed on male *Kptn*^−/−^ mice and *Kptn*^+/+^ controls (*n* = 5 each). (**A**) Significant changes in inter-landmark distances are indicated with lines (colours represent bins of % change from wild-type) for height, length and width of the brain cavity of *Kptn*^−/−^ animals (*P* < 0.05, two-tailed Student's *t*-test, Supplementary Table 1). (**B**) Sagittal sections of 3D reconstructions comparing skull height along the rostro-caudal extent of the brain cavity (arrowheads). (**C**) Anterior-facing coronal sections from representative individual 3D reconstructions to highlight changes in the dorsal curvature (yellow arrows) of frontal and parietal bones in *Kptn*^−/−^ mice (left, green = *Kptn*^+/+^, right, red = *Kptn*^−/−^), and the width of the brain cavity (white arrow, +2.56%, *P* = 0.0356). (**D**) Representative hemisections of male *Kptn*^+/+^ control (*left*) and *Kptn*^−/−^ mutant brains (*right*). (**E**) Volumetric measurements from MRI of female and male *Kptn*^−/−^ mutants and *Kptn*^+/+^ controls comparing total intracranial volume at 16 weeks of age (*n* = 8 per group, two-tailed Student's *t*-tests). Values are plotted as mean ± standard error of the mean (SEM). (**F**) Representative sections from *Kptn*^+/+^ and *Kptn*^−/−^ cortices stained with cresyl violet. (**G**) Morphometric analyses of histological sections (as in **D** and **F**) of *Kptn*^−/−^ mutant brains plotted as percentage difference of *Kptn*^−/−^ from wild-type mean, to identify significant changes in total brain area (shown for Section 1), cortical (shown for Section 1) and corpus callosum thicknesses (Section 2) (*P*-values as described below, two-tailed Student's *t*-tests, further details in Supplementary Fig. 5 and Supplementary material, File 1). (**H**) Cellular features for each cortical layer (layer I to layer VI) in *Kptn*^−/−^ mutant mice (cell count, cell size, cell density) as well as the corresponding layer area (Supplementary material, File 1) at position Bregma −1.34 mm in male mice, shown as a percentage difference of *Kptn*^−/−^ from wild-type mean. n.s. = no significant change (*P* > 0.05); *P < 0.05; **P < 0.01; ***P < 0.001; ****P < 0.0001.

Applying μCT-based cephalometric analysis to examine skull size, we detected an increase in the height, length and width of the brain cavity of the skulls of adult *Kptn*^−/−^ mice (Fig. 4A–D, Supplementary Table 1 and Supplementary Fig. 3). The height of the skull at the midline is consistently elevated throughout the rostro-caudal extent of the brain cavity (5.2% at Bregma/L1-L7, 4.7% at Lambda/L2-L5, *P* < 0.01) as seen in a sagittal resection of representative 3D volumes (Fig. 4B). In coronal resections, the height changes are due primarily to an increase in the dorsal curvature of the frontal and parietal bones (Fig. 4C), rather than an increase in the dorsoventral length of the lateral skull wall. This dorsal bulging is consistent with the changes observed in frontal bossing in humans, where the frontal bone may expand to accommodate pathological brain overgrowth.^65,66^ Applying MRI tensor-based morphometry followed by voxel-based quantification of brain volume,^38^ we detected a 7.7%/9.2% increase (female/male, *P* < 0.001) in mean intracranial volume in *Kptn*^−/−^ mice at 16 weeks when compared to wild-type controls (Fig. 4E). Together, these bone and soft tissue findings indicate that *Kptn*^−/−^ mice exhibit both skull shape abnormalities, including overgrowth and deformation, and megalencephaly. These are consistent with the macrocephaly and frontal bossing seen in KRD.

We next characterized the megalencephaly using previously published histomorphometric methods,^39,40^ quantifying neuroanatomical features across the same three coronal brain regions (Fig. 4D and Supplementary Figs 4 and 5). Substantial brain size anomalies were identified in both adult female and male *Kptn^−/−^* mice at 16 weeks when compared to matched wild-types, with 34 of 78 parameters affected (Fig. 4F, Supplementary Fig. 5A–C and Supplementary material, File 1A–C). The total brain area in both sexes was significantly increased across the three coronal sections (+7.9–14.4%, *P* ≤ 0.0007), concomitant with enlarged cortices in all areas measured, and enlarged corpus callosum structures (Fig. 4F–H). The lateral ventricles were the only brain regions exhibiting a decreased size (section 1: −36%, *P* = 0.05; section 2: −54%, *P* = 0.02), a common observation in mouse and human macrocephaly ascribed to compensatory shrinkage due to increased grey and white matter volume constrained within the skull. Strikingly, we detected no significant difference in hippocampus-related size parameters (Fig. 4G, Supplementary Fig. 5A–C and Supplementary material, File 1A–C). The histological findings are consistent between sexes (Fig. 4G, Supplementary Fig. 5A–C and Supplementary material, File 1A–C).

### Hypercellularity contributes to brain enlargement in KRD

To better understand the underlying cause of the brain enlargement in KRD, we further quantified cortical layer areas, total cell counts and total cell areas (area occupied by cell bodies). All six cortical layers showed an increase in area, largely accounted for by equivalent increase in the number of cells, notably in layers II to V (layer II/III: +21.7%, *P* = 0.0289; layer IV: +20.1%, *P* = 0.0085; layer V: +16.3%, *P* = 0.0281) in *Kptn*^−/−^ compared to wild-type mice (Fig. 4H). Conversely, when looking at cell density and cell size across individual layers of the cortex we found no significant changes in cell density, while mean cell size is not significantly changed apart from layer VI (Fig. 4H): an 8.8% increase in cell size in this layer suggests some contribution of increased cell size to the overall increase in brain size.

The increase in layer size, with concomitant increases in cell numbers, and only limited evidence for a change in cell size, suggest that the underlying mechanism of brain enlargement in KRD is predominantly hypercellularity rather than a change in cell body size (Supplementary material, File 1D), in contrast to what has been observed in some models of mTOR-related macrocephaly.^67^

To better understand the developmental trajectory of brain overgrowth in our mouse model, we compared histomorphology at birth (postnatal Day P0), P20 and 16 weeks of age. P0 allows us to assess whether *Kptn* mutant animals are born with enlarged brains, or conversely overgrow postnatally. P20 represents the stage at which wild-type mice reach 90–95% of adult brain weight^68,69^ and thus allows us to distinguish between an increased growth rate and an extended brain growth period. In the former, we would anticipate a larger brain by the end of normal brain growth (P20), while the latter would lead to later appearance of overgrowth (16 weeks). Using histological methods described above, we detected no phenotypic differences at P0 in the vast majority of parameters (51 of 53) including the total brain area (Supplementary Fig. 5E and Supplementary material, Files 1E and F), indicating that the megalencephaly phenotype is associated with postnatal developmental processes. Interestingly, two parameters were altered at P0, the total area of the hippocampus is reduced by 11% in the *Kptn*^−/−^ mice (*P* = 0.04), and the internal capsule increased by 9% (*P* = 0.03), suggesting that some morphological anomalies originate from prenatal stages but these are highly tissue restricted (Supplementary Fig. 5E and Supplementary material, File 1E and F).

**Figure 5:**
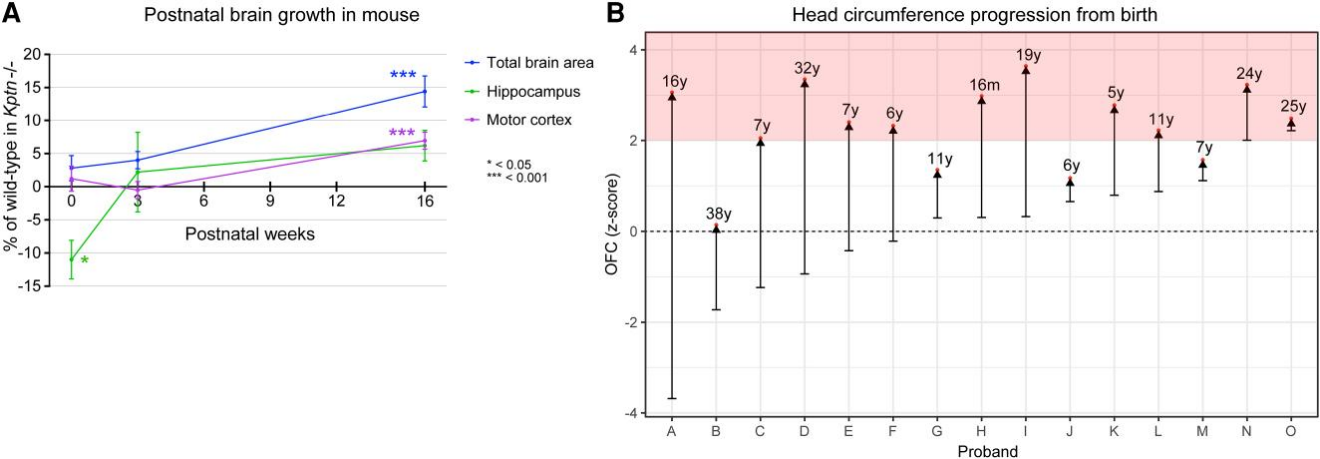
**Assessment of postnatal brain overgrowth in mouse KRD model and human KRD probands**. (**A**) Percentage change in mean of total brain area, motor cortex and hippocampus in *Kptn*^−/−^ mutant mice compared to wild-types at birth, 3 weeks and 16 weeks postnatally, with significance level indicated by asterisks in graph. Points without asterisks are not significantly different (*P* > 0.05, two-tailed Student's *t*-test). (**B**) Occipital frontal circumference (OFC) measurements from 15 individuals with KRD from birth (lower bars) to age at last assessment (red dots). Pink shading in **B** indicates the macrocephalic range, with SD >2.

A similar analysis on P20 mice again identified very few statistically significant changes in regional brain size (Supplementary Fig. 5D and Supplementary material, File 1G), despite a broad upward trend. Consistent with all stages examined, the ventricles of P20 *Kptn*^−/−^ mice are reduced in size, supporting the idea that there is ongoing, but mild overgrowth at early stages that continues into adulthood, eventually resulting in severe megalencephaly. It is striking that *Kptn*^−/−^ mice lack significant overgrowth at P20, when wild-type mice will have reached the end of the normal brain growth. The progressive nature of the disorder is well illustrated when we plot measurements of total brain area, motor cortex and hippocampus with time (Fig. 5A). We note that the underdevelopment of the hippocampus at P0 recovers with age, but never reaches statistically significant overgrowth during the life of the mouse. Taken together with the significant brain size changes in adult *Kptn*^−/−^ mice, these data indicate that the megalencephaly seen in the *Kptn* model is postnatal, progressive and may result from an extended period of brain growth.

### Macrocephaly in humans with *KPTN*-related disorder is postnatal and progressive

We set out to clarify whether the macrocephaly seen in individuals with KRD is also postnatal and progressive. This could provide opportunities to intervene in the disorder progression after birth, thus increasing the feasibility of any proposed treatment. We identified a total of 35 patients with a confirmed molecular diagnosis of KRD (Supplementary material, File 2), including 20 new Amish and non-Amish individuals through our international collaborators, revisited the nine affected individuals originally described from the Amish community, and reviewed OFC data for six from previously published studies.^27-29,31^ Figure 5B shows the OFC trajectories of 15 KRD patients from birth to their most recent available assessment. The majority of KRD patients are born with OFCs within the normal range, and progress with age to become macrocephalic (>2 SD, pink zone in Fig. 5B), with the most rapid increase occurring within the first 2 years of life (representative OFC graph in Supplementary Fig. 6). These data demonstrate further consistency between the human disorder and our mouse model, which both experience progressive postnatal overgrowth.

### KPTN regulates mTOR signalling *in vivo*

**Figure 6:**
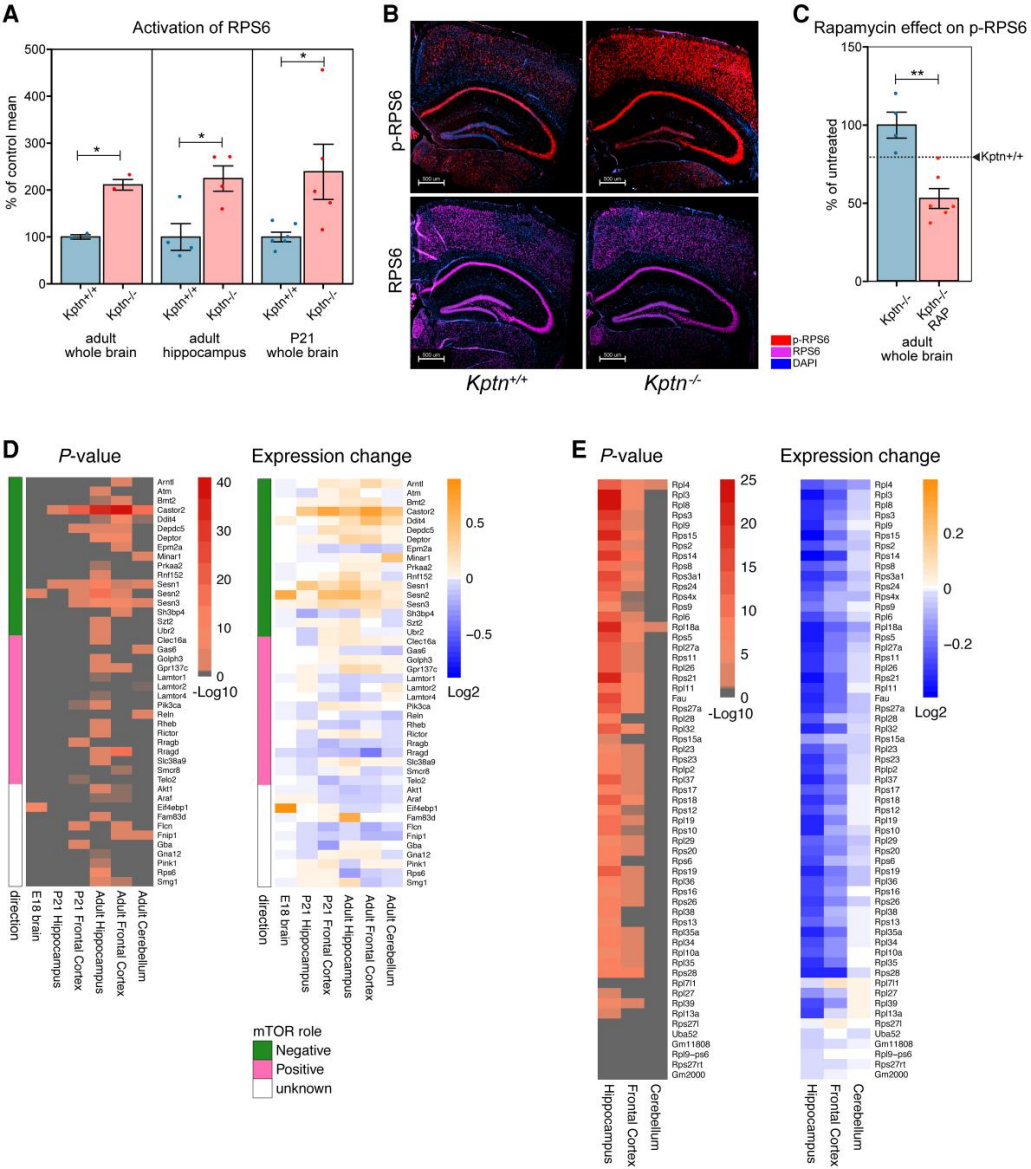
**Assessing Kptn loss-of-function effects on mTOR signalling**. (**A**) Quantification of the phosphorylation of ribosomal protein S6 (RPS6) in wild-type controls (*Kptn*^+/+^) and mutant animals (*Kptn*^−/−^) to test for alterations in mTOR signalling in adult whole brain (*P* = 0.0119, *n* = 2 per genotype), hippocampus (*P* = 0.0189, *n* = 4 per genotype), and in whole brains of juvenile mice at P21 (*P* = 0.0308, *Kptn*^+/+^, *n* = 6; *Kptn*^−/−^, *n* = 5), measured as the ratio of phosphorylated RPS6 (p-RPS6) to total RPS6 signal (units as % of control mean). All *P*-values are from two-tailed Student's *t*-tests. (**B**) Immunostaining for RPS6 (purple) and its phosphorylated form (p-RPS6, red) to reveal changes in cortical and hippocampal activation of the mTOR pathway in *Kptn*^−/−^ mice (nuclei stained with DAPI, blue). (**C**) Quantification of the phosphorylation of RPS6 in *Kptn*^−/−^ animals after 3 days of treatment with vehicle (*Kptn*^−/−^) or rapamycin (*Kptn*^−/−^ RAP) to detect changes in p-RPS6 levels upon treatment (*P* = 0.00188, *Kptn*^+/+^*n* = 4; *Kptn*^−/−^*n* = 6, two-tailed Student's *t*-test). Values are plotted as mean ± SEM, as a percentage of vehicle treated *Kptn*^−/−^ mice. Wild-type levels are indicated by the dashed line and arrowhead (*Kptn*^+/+^). (**D** and **E**) Bulk RNA-Seq reveals transcriptional changes to mTOR pathway components (**D**) and downstream ribosomal gene network (**E**) in *Kptn*^−/−^ mice at embryonic and postnatal stages as indicated, compared to *Kptn*^+/+^ controls. Statistically significant (α < 0.05) Log_2_-fold changes in expression are indicated by non-grey adjusted *P*-value heat map cells. Role in mTOR pathway is indicated as green/pink/white colour scheme in **D**. All western blot images can be found in Supplementary material, File 5.

KPTN functions in a protein complex (KICSTOR) negatively regulating mTOR activity in response to nutrient availability.^18,32^ Given the known contribution of mTOR pathway members in numerous brain overgrowth disorders in humans,^9,70-72^ this provides a plausible mechanism for KRD overgrowth. To examine whether KPTN modulates mTORC1 signalling in our *in vivo* model, we performed western blot assays on brain tissue from *Kptn*^−/−^ and *Kptn*^+/+^ mice assessing ribosomal protein S6 (RPS6), a known downstream target of the mTORC1 pathway whose phosphorylation state is strongly linked to pathway activation.^73,74^ Our results in both whole brain and hippocampus of adult animals revealed a significant increase in mTOR pathway activity in *Kptn^−^*^/−^ mice, as indicated by increased phosphorylation of RPS6 (*P* < 0.05; Fig. 6A). Importantly, we found that mTOR activation is present at P21 (*P* = 0.0308, Fig. 6A), the stage at which the *Kptn^−^*^/−^ model experiences brain overgrowth. Immunostaining for phosphorylated RPS6 (p-RPS6) in histological sections of brain tissue highlights the widespread cortical and hippocampal activation of mTOR activity in *Kptn^−^*^/−^ mice (Fig. 6B). To confirm that the increase in p-RPS6 caused by loss of *Kptn* is mTOR-dependent, we treated a cohort of mice with rapamycin, an mTOR pathway inhibitor.^75-77^ We found that the excess p-RPS6 signal seen in *Kptn^−^*^/−^ animals is significantly reduced after 3 days of treatment (to well below wild-type levels), confirming the presence of hyperactive mTOR signalling in our model (*P* = 0.00188, Fig. 6C). These data are consistent with the proposed role of Kptn as a negative regulator of mTORC1 activity and provides *in vivo* evidence of a role for mTOR signalling in KRD.

### Transcriptional changes in *Kptn* animals and human neural cells highlight mTOR pathway dysregulation

While few transcriptional readouts have been described for mTOR activity, the tight regulation of this critical metabolic pathway involves extensive use of feedback loops to maintain homeostasis.^78^ We hypothesized that the loss of negative mTOR regulation in our *Kptn^−^*^/−^ model would likely result in detectable changes to the expression of these mTOR pathway members, and genes involved in the downstream cellular processes. We performed RNA-Seq on dissected P21 and adult brain tissues (prefrontal cortex, hippocampus and cerebellum), comparing gene expression between *Kptn^−^*^/−^ and *Kptn*^+/+^ animals. Extensive dysregulation of both positive and negative regulators of the mTOR pathway were observed (Fig. 6D). Interestingly, adult hippocampus and cortex show strong dysregulation, concordant with the immunostaining results in adult tissue. A notable observation is the enrichment in upregulation of negative mTOR regulators (Fig. 6D), likely reflecting a strong negative feedback response in an attempt to attenuate the hyperactivated mTOR signalling resulting from loss of KPTN protein. Numerous genes involved in protein synthesis, including most ribosomal protein genes, are dysregulated in the *Kptn^−^*^/−^ mice (Fig. 6E and Supplementary material, File 3A). These two observations are supported by the statistically significant enrichment of differentially expressed genes related to these processes, when assessed by unbiased GO enrichment^46^ (Supplementary material, File 4A). When we test specifically for dysregulation of mTOR pathway genes, five of six tissues examined show a significant enrichment (hypergeometric test *P* < 0.05, Supplementary material, File 4B). These data are concordant with the known regulation of ribosomal protein genes by mTOR and its critical role in controlling protein synthesis.^79-81^

To confirm the relevance of our mouse findings to human biology, we generated human iPSC models of KRD. We selected a gRNA targeting exon 8 of the human *KPTN* gene, binding directly between the coordinates of two *KRD*-associated variants, and aiming to recapitulate the known nonsense variant (p.Ser259*).^27^ Using CAS9 riboprotein electroporation in the KOLF2C1 cell line,^41^ we generated clones carrying frameshifting indels in exon 8 of *KPTN* (Supplementary Fig. 1A). To examine the effect of *KPTN* LoF in a biologically relevant cell type, we differentiated our iPSC models into PAX6-positive cortical NPCs using established methods.^42^ The functional consequence of the frameshifting variants was characterized by RNA-Seq, which reveals a dose-dependent reduction in detectable *KPTN* transcript reads in heterozygous (41% reduction) and homozygous (89% reduction) mutant NPC lines (Supplementary Fig. 1C), likely reflecting robust nonsense-mediated decay.

**Figure 7:**
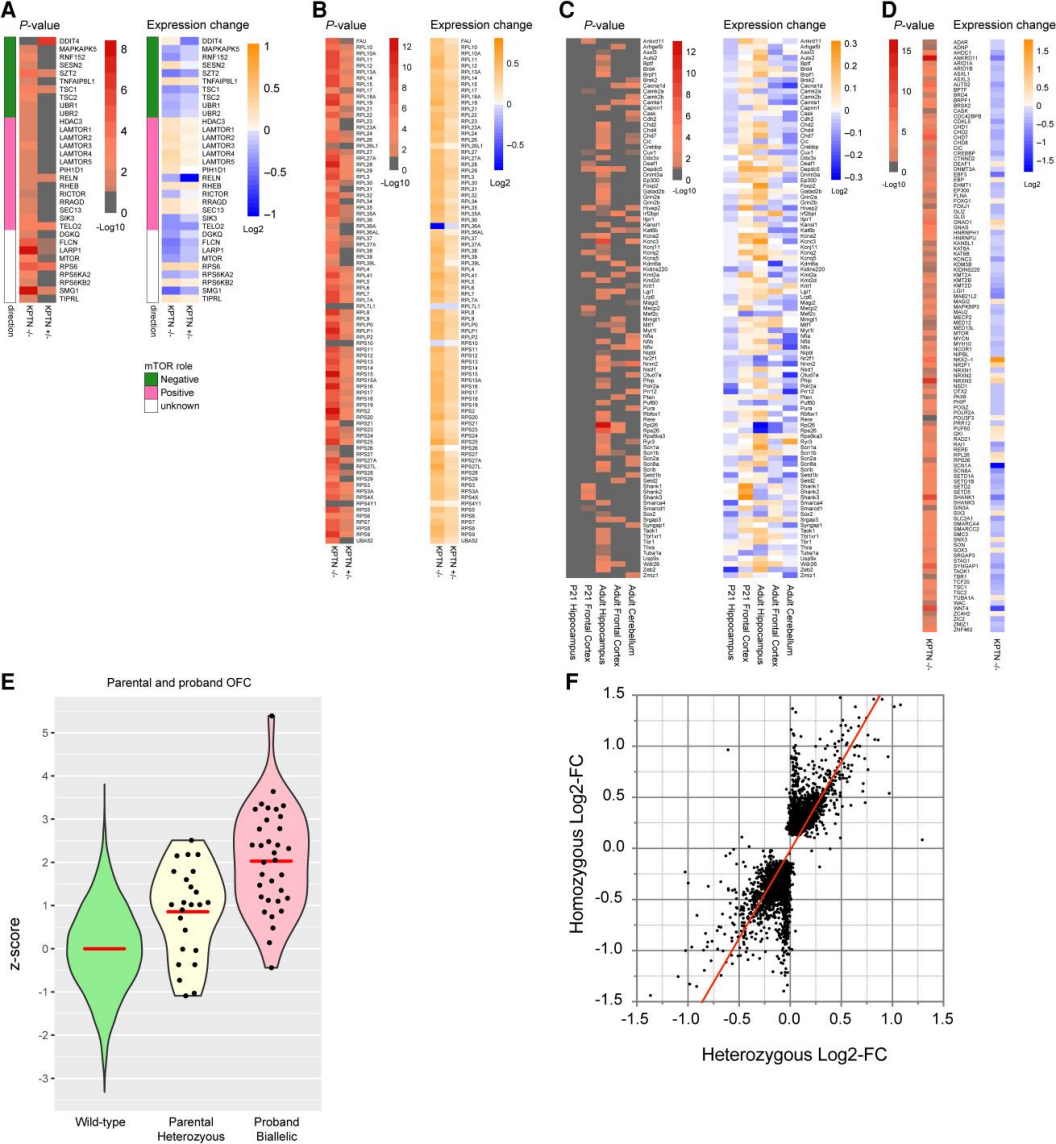
**Assessing transcriptional changes in mTOR pathway and developmental disorder genes in human neural stem cell model of KRD, and morphological correlates of *KPTN* dosage sensitivity**. (**A** and **B**) Transcriptional changes in mTOR pathway components (**A**) and downstream ribosomal gene network (**B**) in KPTN LoF NPC models compared to wild-type controls. Heterozygous *KPTN* LoF cells (*KPTN*^+/−^) were included to look for the presence of any intermediate mTOR pathway phenotypes consistent with dosage sensitivity to loss of *KPTN* expression. (**C** and **D**) Dysregulation of dominant haploinsufficient developmental disorder associated genes in mouse brain (**C**) and human neural cell models of KRD (**D**). Statistically significant Log_2_-fold changes in expression are indicated by non-grey adjusted *P*-value heat map cells. (**E**) Occipitofrontal circumference (OFC) measurements in heterozygous parents (Parental Heterozygous) of KRD probands (mean *z*-score = 0.856, *P* = 0.000028, one sample, two-sided *z*-test against wild-type distribution, *n* = 24) alongside the wild-type OFC distribution (Wild-type) and that of affected individuals with biallelic *KPTN* variants (Proband Biallelic, as detailed in Supplementary material, File 2; Proband, mean *z*-score = 2.027, *P* < 0.00001, one sample, two-sided *z*-test against wild-type distribution, *n* = 35). (**F**) Comparison of normalized read counts from RNA-Seq on *KPTN* LoF NPC models to examine correlation between the gene dysregulation in homozygous and heterozygous *KPTN* LoF cell lines (Pearson *r* = 0.759, *P* < 0.0001).

Transcriptomic analysis of bulk RNA-Seq of these human brain stem cell models of KRD and isogenic wild-type controls (Supplementary material, File 3B) reveals significant differential expression of numerous genes involved in mTOR signalling, and nearly complete dysregulation of the ribosomal protein gene network (Fig. 7A and B). The tuberous sclerosis complex (TSC) proteins, important inhibitors of the mTOR pathway, are both downregulated in *KPTN*^−/−^ NPCs. LoF variants in TSCs are associated with tuberous sclerosis complex, a multi-system tissue overgrowth syndrome affecting brain, skin, kidney, heart and lung.^82^ We found that 36% of genes dysregulated in *TSC2*^−/−^ neurons^83^ (Supplementary material, File 3C) are also differentially expressed in our *KPTN*^−/−^ NPCs (301 of 834 expressed in NPCs, 1.35-fold over-enriched, hypergeometric test *P* = 4.3 × 10^−10^), thus identifying a common signal in neural cell types driven by convergent disease mechanisms affecting the mTOR pathway.

The connectivity map^84^ (CMAP), a large-scale collection of transcriptional response profiles after compound exposure (20 000 different compounds and drugs) or gene knockdown across numerous cell lines, uses negative correlation between drug-disease profile pairs to identify potential therapeutics to reverse disease states. We queried the CMAP database with the transcriptional response signature of our *KPTN*^−/−^ NPCs. We found that torin-2,^85^ a known mTOR inhibitor, scores as the most strongly negatively correlated profile (global correlation score of −96.28 across all cell lines) (Supplementary Fig. 7A), and is therefore considered the most promising treatment for the queried disease profile. Interestingly, in HCC515 cells, five of the top 32 anti-correlated signatures are mTOR inhibitors (torin-2, QL-X-138, torin-1, sirolimus and WYE-354) with scores between −99.56 and −93.23 (Supplementary Fig. 7B). This strong anticorrelation with mTOR inhibitor responses, detected in non-neural cancer lines, suggests that the effect of loss of KPTN protein on the mTOR pathway is robust and specific.

### Increases in radial glial and intermediate precursor marker expression

It has been shown that the mTOR pathway is both active in and critical for the development and differentiation of neural stem cells in both mouse and humans.^21,22,86^ Pathological increases in mTOR signalling, seen in tuberous sclerosis complex and somatic *MTOR* variants linked to hemimegancephaly,^24,25^ point to a significant input of the mTOR pathway in the control of neural stem cell expansion and differentiation. To understand how the loss of KPTN protein is leading to hypercellularity in KRD, we examined the expression of markers of neural stem cells and radial glia, and those of proliferating neuronal progenitors in our mouse model. We found significant increases in levels of radial glial markers (e.g. nestin, vimentin, GLAST/*Slc3a1*, and *Thrsp*) and intermediate progenitor markers (e.g. *Tbr1*, *Eomes*/Tbr2, *Neurod2*, *Btg2*)^87-91^ in the mouse brain at both P21 and adult stages (Supplementary Table 2). These striking increases in gene expression provide preliminary supportive evidence for the model in which the persistence, beyond P21, of excessive neural stem cells and cycling neural progenitors contribute supernumerary neuronal cells, resulting in significant pathological brain overgrowth. Further study will be required to determine whether these data indeed underlie the cellular basis of megalencephaly in KRD.

### Epilepsy gene networks in *KPTN*-related disorder

Around half (47%) of KRD patients experience seizures, either generalized tonic-clonic seizures alone, or with absence and/or complex partial seizures.^27-31^ The onset of seizures can occur at any age in both children and adults and seizures are often refractory to treatment requiring polytherapy. Although we did not observe any clinical seizures during the maintenance and testing of *Kptn^−^*^/−^ mice, the absence of detectable spontaneous seizures in our model is not totally unexpected. Other mouse models of human mTOR-associated epilepsies such as *SZT2*^32^ (a KPTN binding partner and mTOR regulator) show a reduced threshold for seizures after pentylenetetrazole and electroconvulsive treatment, while lacking the spontaneous seizures observed in the corresponding human disorder.^15,16^ Transcriptomic analysis of our KRD models, however, reveals the dysregulation of numerous known epilepsy-associated genes^92,93^ (syndromic and non-syndromic) in both mouse brain (290 of 844 expressed, Supplementary Fig. 8A) and cortical NPCs (281 of 807 expressed, Supplementary Fig. 8B). Among the differentially expressed seizure genes, around half of the mouse genes (*n* = 122) and a third of the human genes (*n* = 97) are concordant between direction of change and known mechanism of disease (e.g. downregulated expression of a LoF mechanism disease-associated gene). There is a significant overlap in differentially expressed seizure genes between mouse and human KRD models (107 genes in common, 1.5-fold over-enriched, hypergeometric test *P* = 3.6 × 10^−7^) (Supplementary Fig. 8C). Of particular interest are genes known to be dosage-sensitive and are therefore most likely to contribute to seizure predisposition. For example, *SCN1A*, *SCN8A*, *SLC2A1*, *SYNGAP1*, *SHANK1* and *LGI1* all show >30% reduction in expression in *KPTN*^−/−^ NPCs. *KCNQ3*, a potassium channel protein found mutated in neonatal seizures, shows a 25% increase in expression in these cells, consistent with the suspected altered-function mechanism in patients.^94,95^ Of others known to operate by an altered or gain-of-function mechanism, *Sik1* is of note, with a 90% increase in expression in the prefrontal cortex of *Kptn*^−/−^ mice (*P*-adj = 0.0012). Patients carrying *SIK1* missense mutations experience severe developmental epilepsy, with mutations altering the protein's ability to correctly regulate MEF2C activity in neurons.^96,97^ Taken together, these data suggest that KPTN LoF is resulting in a dysregulation of numerous genes that control electrophysiological homeostasis in brain tissue, potentially leading to a susceptibility to seizures in affected individuals.

### Dosage sensitive gene networks are disrupted in KRD models

The regulation of mTOR signalling is critical to properly regulated growth and differentiation during development.^11,98^ The loss of an important negative regulator of this pathway during development would therefore be expected to disrupt important gene networks that regulate these processes. Indeed, we observed that numerous genes causally linked to other neurodevelopmental disorders are differentially expressed in our KRD models. Haplo-insufficient genes (dosage-sensitive disease-associated genes, Fig. 7C and D), whose phenotype appears in heterozygous LoF, are key regulators of brain development and normal brain function.^99^ We found a surprisingly consistent downregulation of these (82% of 111 genes, Fig. 7D) in *KPTN*^−/−^ human cortical neural precursors (*P* < 0.00001, Fisher's exact test). This includes a >25% reduction in expression of important chromatin-modifying proteins and transcriptional regulators *AUTS2*, *AHDC1*, *ANKRD11*, *CHD7*, *CREBBP*, *CHD2*, *EBF3*, *KMT2D*, *TBR1*, *TCF20*, *SETD1B* and *ZIC2*. Individual disruptive variants in each of these genes results in severe neurodevelopmental deficits in KRD-relevant phenotypic areas (Supplementary Table 3). Substantial changes in the expression of these proteins indicates a significant impact of *KPTN* gene function on a broad network of developmentally important genes, whose reduced expression has a known impact on cognitive function.

### Human brain growth is sensitive to KPTN dosage

During clinical data collection from Amish individuals affected by KRD, it was observed that several of their parents presented with larger than average head size. As these parents are heterozygous carriers of pathogenic *KPTN* gene variants, we carried out a systematic analysis of OFC in 24 parents of identified KRD cases. We observed a significant increase in head size (mean *z*-score = 0.856, *P* = 0.000028) in carriers compared to the normal population distribution, though infrequently in the macrocephalic range (4 of 24 had an OFC >2 SD, Fig. 7E). This surprising observation suggests that while KRD is a recessive condition, *KPTN* alleles have an additive effect on head size, with reduced KPTN dosage in heterozygous carriers leading to head overgrowth, possibly due to the same hypothesized mechanism of mTOR hyperactivation. To test this directly, we differentiated heterozygous *KPTN* LoF iPSCs into cortical neural precursors. RNA-Seq on these cells revealed a strong correlation between the gene dysregulation in homozygous and heterozygous *KPTN* lines (Pearson *r* = 0.759, *P* < 0.0001, Fig. 7F). We found an intermediate mTOR pathway dysregulation in the heterozygous cells, and widespread disruption to the ribosomal protein gene network, consistent with that observed in the homozygous *KPTN* lines (Fig. 7A and B and Supplementary material, File 3B). These data strongly suggest that human head size is sensitive to *KPTN* gene dosage, likely through its action within the mTOR pathway. Although none of the heterozygous carrier parents had any reported cognitive impairment or history of seizures, further studies will be required to understand whether the growth effect is accompanied by any changes in neurological function.

## Discussion

We have generated and characterized two informative models of *KPTN*-related disorder, a recent addition to the growing family of mTORopathies. Through behavioural, cognitive, morphological and molecular investigations we have demonstrated robust concordance between the *Kptn*^−/−^ mouse and numerous aspects of human KRD. Our detailed characterization of the progressive nature of brain overgrowth in KRD provides critical insight into disease progression. Through animal and cellular modelling, we have strengthened the link between KPTN and mTOR signalling, and by inference, the role of aberrant mTOR signalling in KRD.

Our preclinical therapeutic investigation indicates that mTOR dysregulation in KRD is rapamycin-sensitive, providing a pathway to possible clinical intervention. The relationship between megalencephaly and cognitive dysfunction in KRD is unclear. The brain overgrowth phenotype could be the primary cause of the cognitive deficits observed in KRD. Alternatively, the neurological phenotypes could be caused by the persistent deregulation of mTOR signalling we observe in adult *Kptn*^−/−^ mice, given the known role of mTOR in cognition and memory formation.^14^ If so, the morphological changes in KRD could simply reflect the effects of *KPTN* variants on brain growth via overactive mTOR signalling. Indeed, the neurological features of KRD are strikingly similar to Smith-Kingsmore syndrome caused by germline gain-of-function variants in *MTOR.*^26^ As mTOR inhibitors are generally well tolerated,^100,101^ the path to clinical intervention in KRD may be tantalizingly short, and follow-up experiments using our mouse and cellular models should provide critical information to shape clinical trials.

Our study highlights the strength of combining clinical investigation with the ongoing characterization of disease models, allowing a more thorough description of both human and model disease pathophysiology. Using our mouse findings in cognitive function, we were able to subsequently identify parallel effects in affected individuals. Our analyses of mouse model head morphology revealed a concordant widespread overgrowth in both brain tissue and skull bones. Through a careful examination of the developmental trajectory of brain overgrowth in our mouse model, we were able to confirm the progressive nature of KRD macrocephaly. Conversely, we were able to take clinical observations from apparently unaffected parents and confirm in cellular models that the heterozygous carriers of pathogenic *KPTN* LoF variants likely experience mTOR dysregulation, contributing to a significant increase in head circumference. While the role of somatic and germline mutations affecting mTOR signalling in pathological brain overgrowth is well established,^102,103^ we were surprised to find such a strong, yet previously unrecognized phenotype effect of brain overgrowth in heterozygotes. There is increasing evidence of a role for recessive disease alleles in causing incompletely penetrant phenotypes in the general population, as the rapidly growing sequencing efforts provide the necessary power to detect these effects.^34-37^ While *KPTN* heterozygotes are not known to experience any neurological consequences, constraint metrics such as loss-of-function observed over expected upper bound fraction (LOEUF)^104^ indicate that *KPTN* LoF alleles may be experiencing purifying selection beyond what would be expected from a purely autosomal recessive disorder gene (observed/expected LoF = 0.5, LOEUF = 0.81). Further investigation into the phenotypic consequences of carrier state will be needed to clarify this apparently benign effect on head size, and indeed whether natural variation in other mTOR pathway genes is detectably contributing to differences in brain growth (as suggested in Reijnders *et al*.^105^).

By exploring the extent and character of transcriptional dysregulation in our KRD models, we identified perturbations to numerous mTOR pathway members, seizure-related genes and transcriptional regulators essential to normal brain development and function. The persistence of progenitor marker genes (for both radial glia and intermediate neuronal precursors) beyond their normal period of expression is consistent with a model in which chronic hyperactivation of the mTOR pathway during postnatal brain development may result in the pathological persistence of proliferating progenitor cells, and the supernumerary differentiation of neural cell types beyond P21. These dysregulated postnatal developmental processes likely contribute to the observed progressive brain overgrowth. The modest evidence we have identified of changes in cell soma size require further investigation, to determine the relative balance of proliferation versus cell enlargement in the KRD-related megaencephaly. A careful examination of the proliferative and morphological profiles of *KPTN* LoF iPSC-derived cells, for example, in the context of brain organoids and cortical neuronal differentiation should provide a more detailed view of the cellular dynamics resulting in macrocephaly in KRD. The structural changes that we and others have observed are likely compounded by chronic mTOR hyperactivation in the adult brain, leading to significant neurocognitive phenotypes in our models and in KRD in humans.

Our multimodal analysis of mouse and cellular models of KRD provides a deeper insight into the underlying mechanisms of disease, and places KRD in the context of a family of disorders with a common underlying mechanism of mTOR dysregulation. Further work to refine our understanding of the timing and affected cell types during postnatal development will contribute to a more informed therapeutic strategy in treating this and other mTOR-related disorders. The promising treatment outcomes of related mTORopathy mouse models^100,106-108^ provide encouraging evidence supporting the aim of effective therapeutic intervention in KRD.

## Supplementary Material

awad231_Supplementary_Data

## Data Availability

The European Nucleotide Archive accession numbers for the RNA-Seq sequences reported in this paper are as follows: Mouse-derived brain RNA-Seq samples: ERA941410-ERA941421, ERA941432-ERA941465, ERA2115013-ERA2115023, ERA2115035-ERA2115045. IPSC-derived NPC samples: ERS8443331, ERS8443330, ERS8443332, ERS8443333, ERS8443322, ERS8443323, ERS8443326, ERS8443327, ERS8443328, ERS8443329.
