# Supplementary material for: Models of KPTN-related disorder implicate mTOR signalling in cognitive and overgrowth phenotypes
Source: Brain. 2023 Jul 12;146(11):4766–83. doi: 10.1093/brain/awad231 (PMC10629792; doi:10.1093/brain/awad231)
Supplement: awad231_Supplementary_Data [file awad231_supplementary_data.zip › brain-2022-02095-File013.pdf]

SUPPLEMENTARY FILE 5

Adult whole brain western blots

Anti-S6

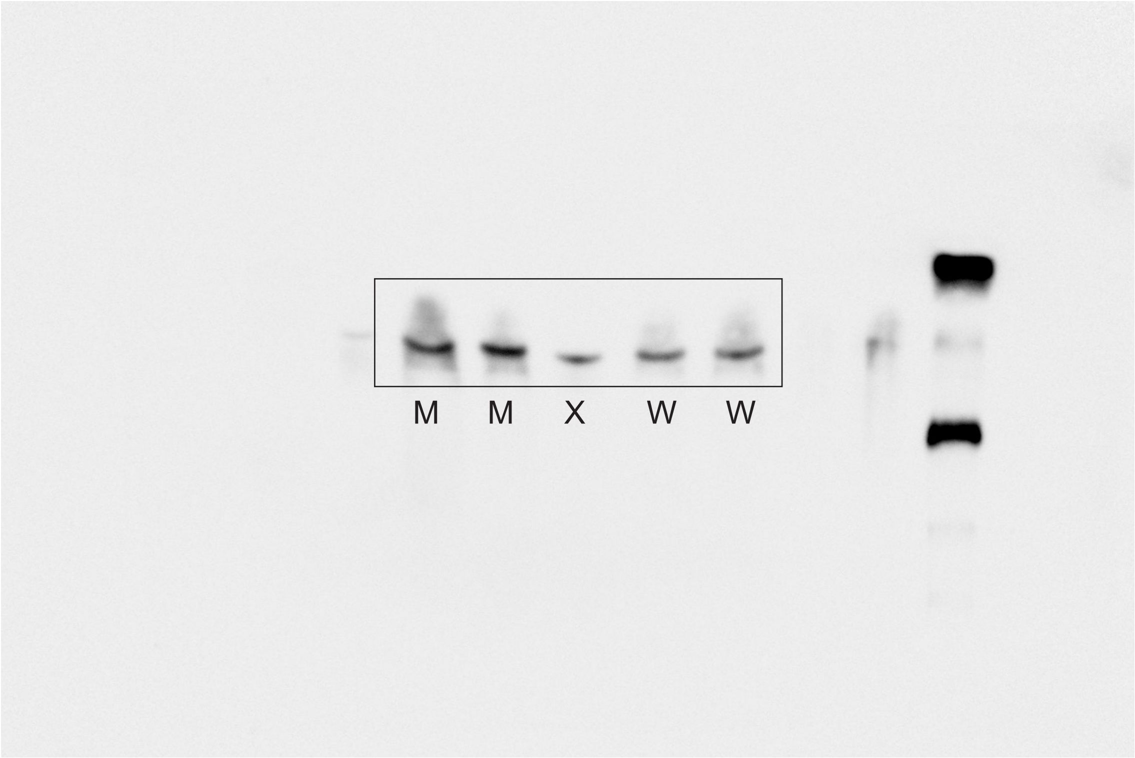

Anti-phosphoS6

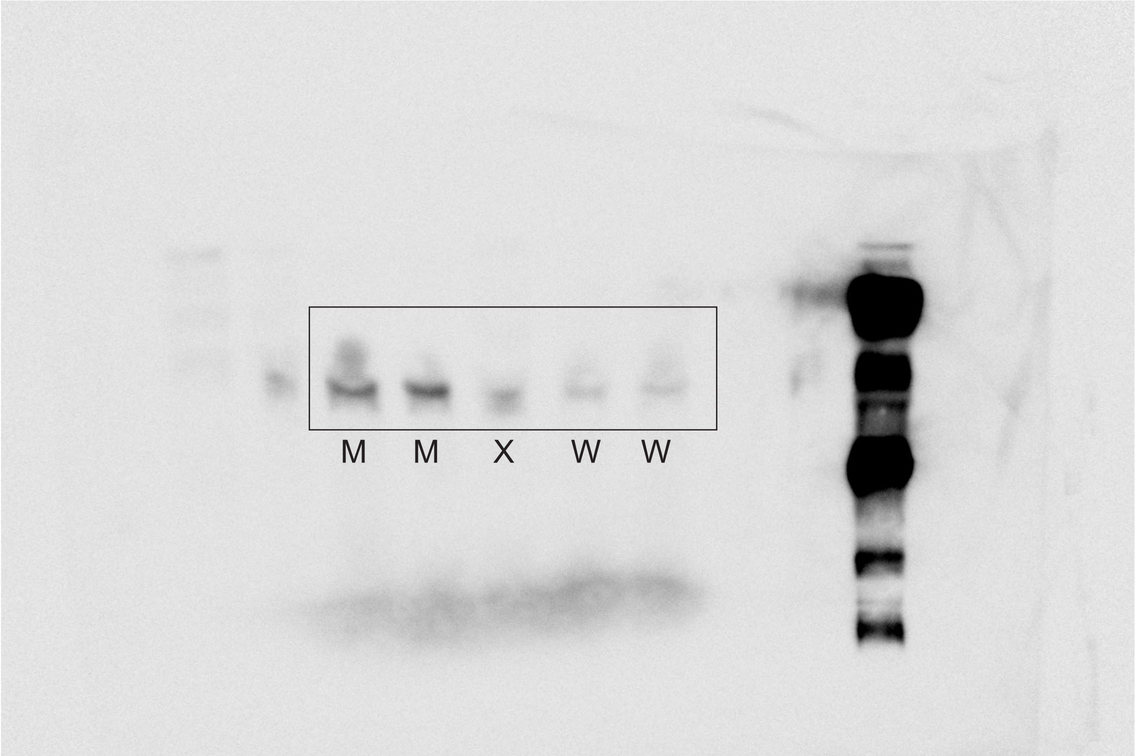

M: *Kptn* -/- mutant
W: *Kptn* +/+ wildtype

Adult Hippocampus western blots

Anti-S6

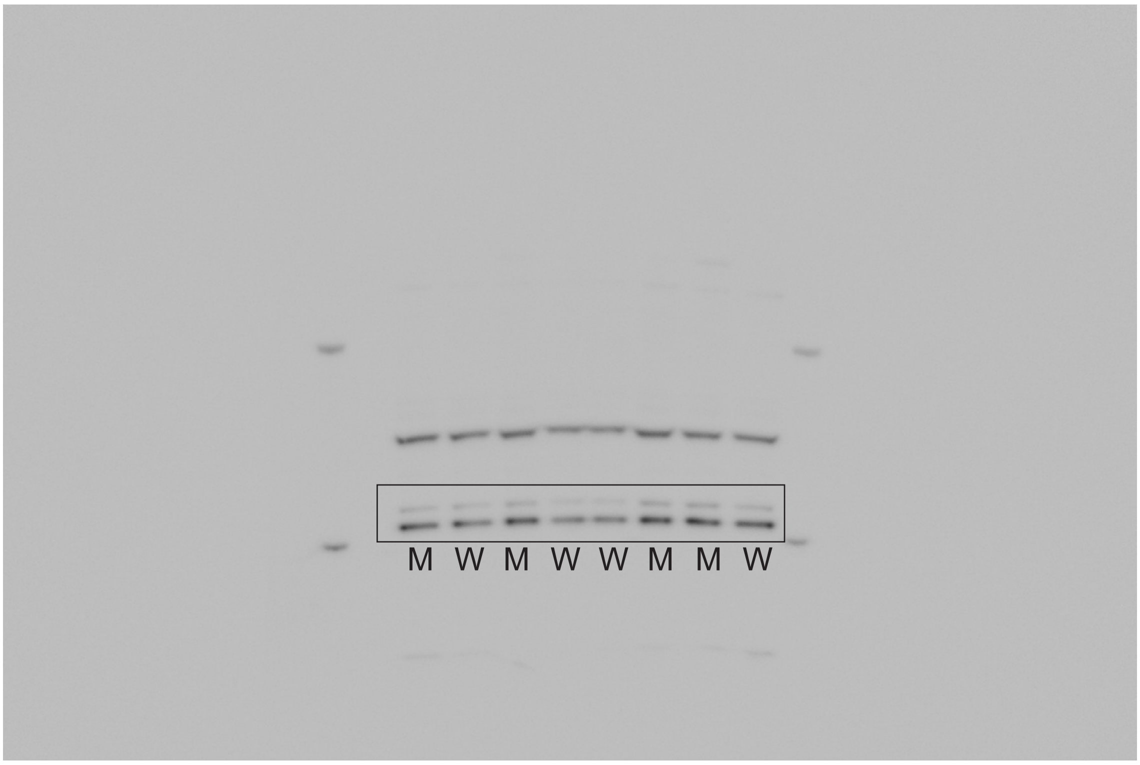

Anti-phosphoS6

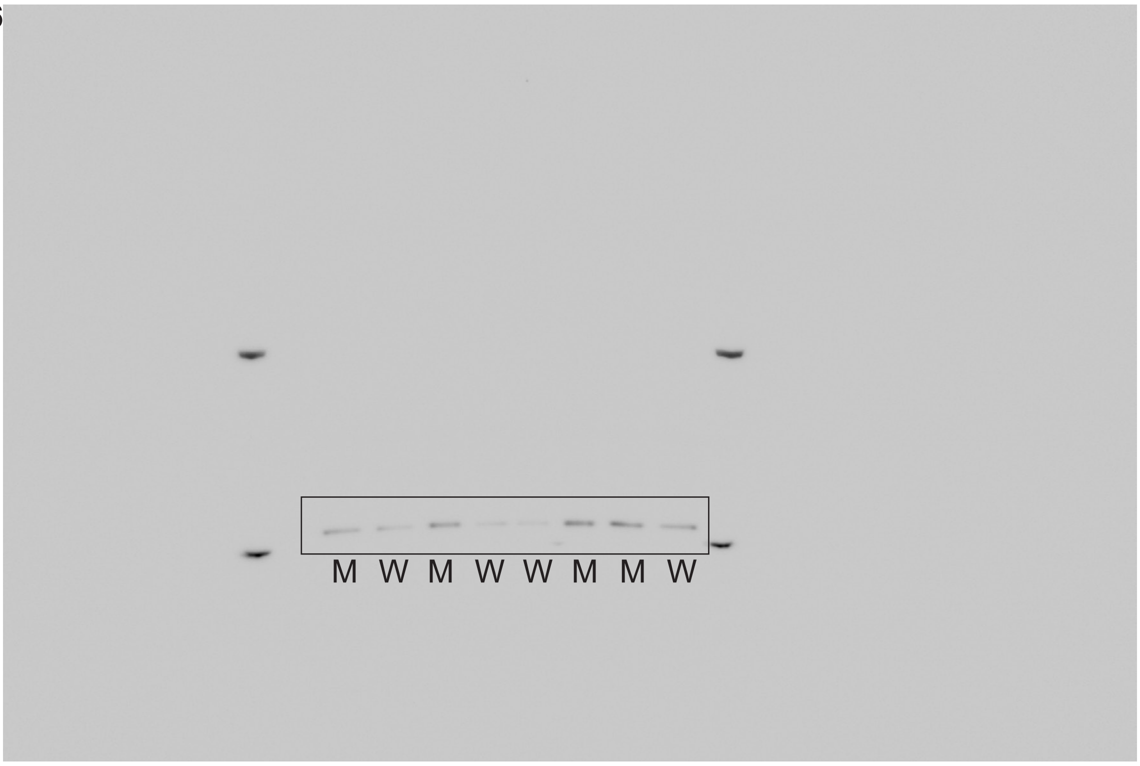

M: *Kptn* -/- mutant
W: *Kptn* +/+ wildtype

P21 whole brain western blots

Anti-S6

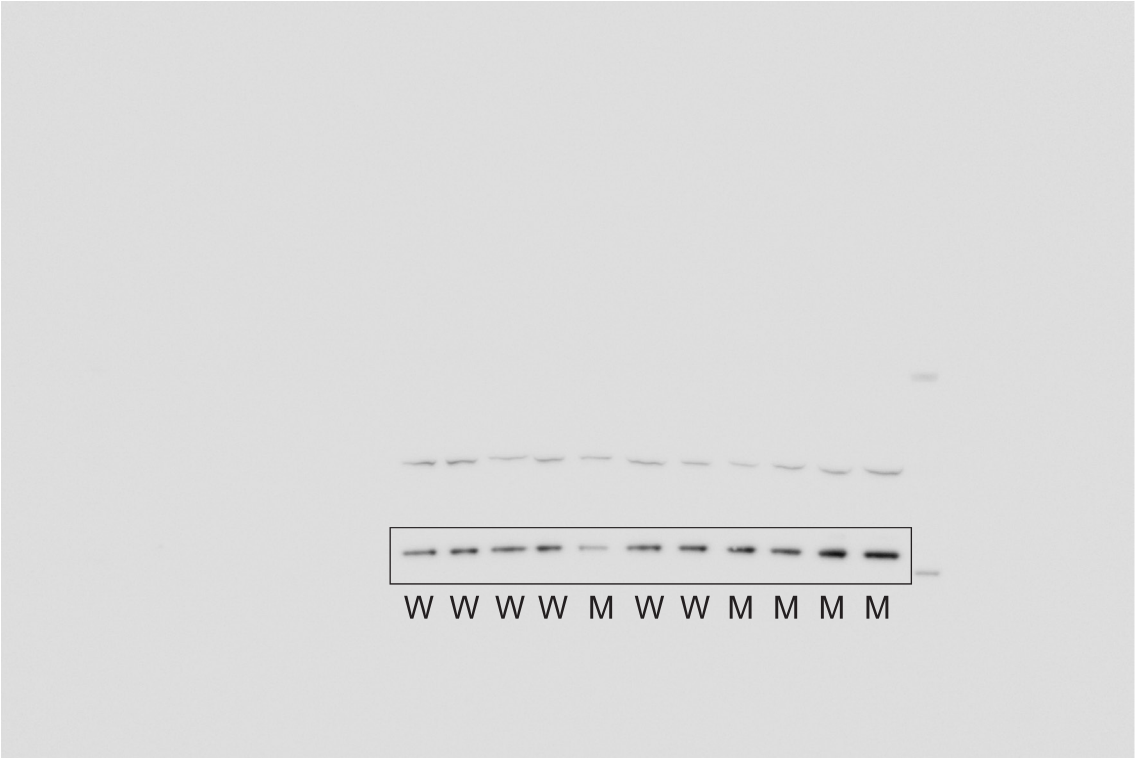

Anti-phosphoS6

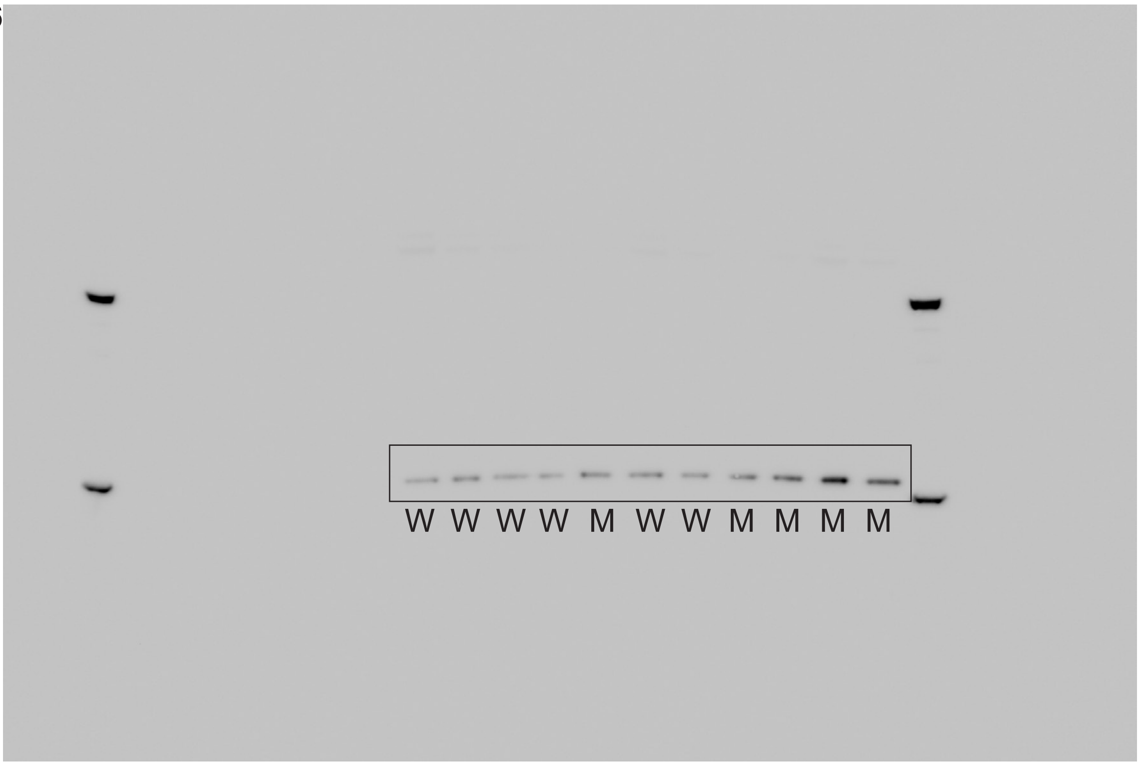

M: *Kptn* -/- mutant

W: *Kptn* +/+ wildtype

Adult Rapamycin treatment Western blots

Anti-S6

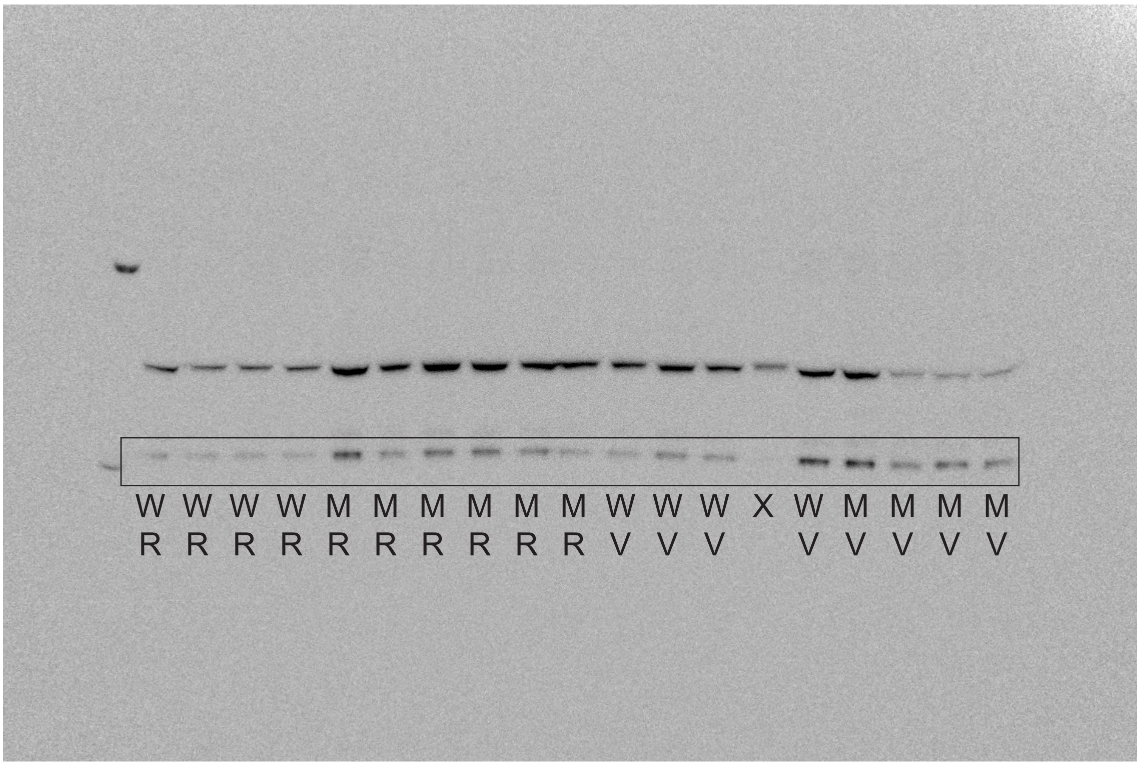

Anti-phosphoS6

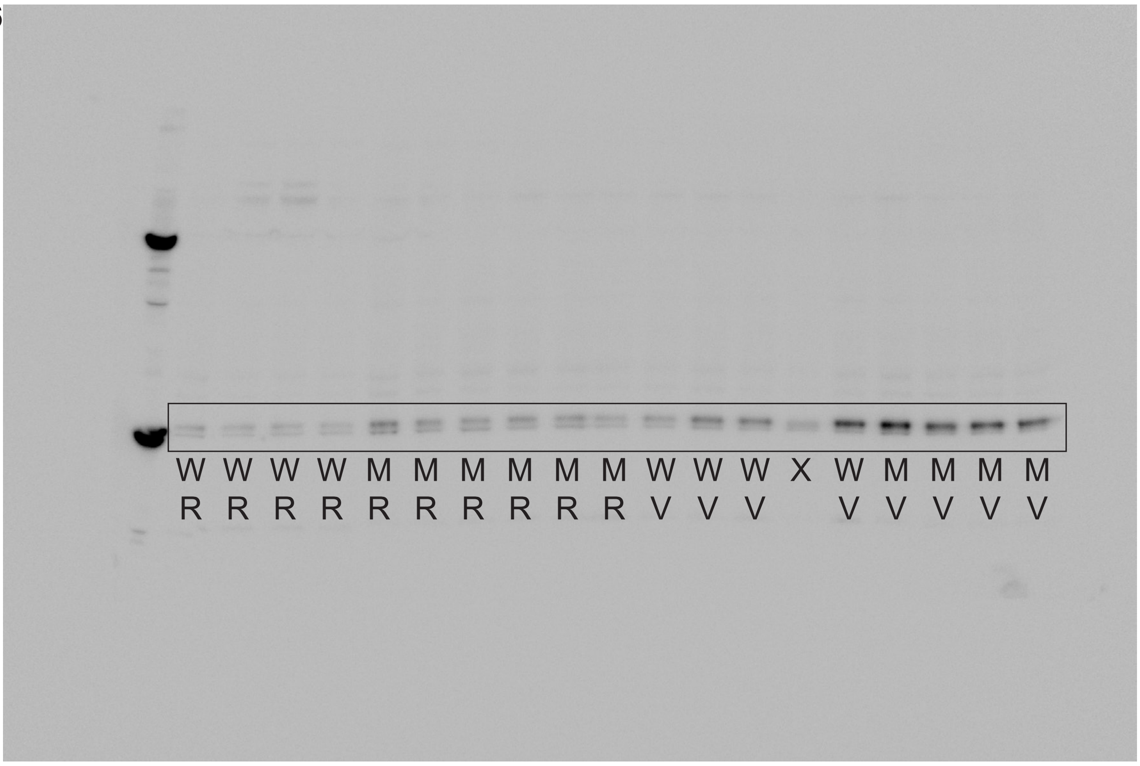

MR: *Kptn* -/- mutant with Rapamycin
MV: *Kptn* -/- mutant with vehicle
WR: *Kptn* +/+ wildtype with Rapamycin
WV: *Kptn* +/+ wildtype with vehicle
