# Supplementary material for: Models of KPTN-related disorder implicate mTOR signalling in cognitive and overgrowth phenotypes
Source: Brain. 2023 Jul 12;146(11):4766–83. doi: 10.1093/brain/awad231 (PMC10629792; doi:10.1093/brain/awad231)
Supplement: awad231_Supplementary_Data [file awad231_supplementary_data.zip › brain-2022-02095-File014.pdf]

LIST OF SUPPLEMENTARY DATA IN THIS PDF

SUPPLEMENTARY METHODS
AUTHOR CONTRIBUTIONS

SUPPLEMENTARY FIGURE1
SUPPLEMENTARY FIGURE2
SUPPLEMENTARY FIGURE3
SUPPLEMENTARY FIGURE4
SUPPLEMENTARY FIGURE5
SUPPLEMENTARY FIGURE6
SUPPLEMENTARY FIGURE7
SUPPLEMENTARY FIGURE8

SUPPLEMENTARY TABLE1
SUPPLEMENTARY TABLE2
SUPPLEMENTARY TABLE3

ADDITIONAL SUPPLEMENTARY FILES (larger multi-tab excel tables)
(legends provided below)

SUPP FILE1 ANATOMY.xlsx
SUPP FILE2 KRD PROBANDS.xlsx
SUPP FILE3 RNASEQ.xlsx
SUPP FILE4 MOUSE PATHWAY ANALYSIS.xlsx
SUPP FILE5 WESTERN BLOTS.pdf

SUPPLEMENTARY METHODS

Mouse production

The mouse model was generated at the Wellcome Sanger Institute. The Kptn$^{tm1a(EUCOMM)Wtsi}$ mice were kept on a C57BL/6NTac background (Taconic Biosciences). The tm1a 'knockout-first' allele was generated by the insertion of an IRES:lacZ trapping cassette and a LoxP flanked promoter-driven neo cassette into an intron, disrupting *Kptn* gene function at the mRNA level by interfering with transcription downstream of the cassette site[1–3].

Housing and breeding of mice and experimental procedures were carried out under the authority of UK Home Office project and personal licenses. All procedures were carried out in accordance with the Animal Welfare and Ethical Review Body of the Wellcome Sanger Institute and the UK Home Office Animals (Scientific Procedures) Act of 1986, under UK Home Office PPL 80/2472 and PPL P6320B89B. Mice were housed in specific pathogen-free mouse facilities with 12 h light/dark cycle. Food and water were available *ad libitum*, except where indicated otherwise.

Mouse behavioural paradigms

In all assays, the mice were tracked using over-head infrared video cameras and a user-independent automated video tracking software Ethovision XT 8.5 (Noldus Information Technology, The Netherlands). All experiments were carried out on mice aged 10-22 weeks. The mice were handled for 2-3 days before the onset of testing. All mice were habituated to the behavioural room in their home cages for ≥ 30 minutes under the same light condition as the test. Male mice were tested unless stated otherwise.

Open field assay

To test for locomotor capabilities, the open field test was run as previously described[4]. In all open field trials, mice were placed in the left corner of the open field arena. Two animals were tested in parallel in two separate open fields (74 cm x 74 cm), for an observation time of 10

minutes. A centre zone was designated with equidistant borders to the arena walls (8 cm). The arenas were cleaned thoroughly with ethanol free wipes to remove odour traces. A period of movement was defined when the mouse reached a velocity of 2 cm/s over two frames; a period of non-movement was defined when velocity was lower than 1.75 cm/s over two frames. Significant differences in distance moved and time spent moving were assessed using a Student's t-test (α=0.05).

Light/dark box assay

The light/dark box assay was used to test for anxiety-related behaviours, as previously described[5,6]. The assay was conducted in a darkened room. After 30 minutes of habituation to the room in their home cages, mice were separated into new cages (with clean bedding, food pellets, cardboard play tunnels, and some used bedding), with two mice per cage. The light/dark box was placed into an open field arena, positioned in the same location in the open field arena in all experiments. The light zone had two spotlights facing down directly into the compartment, illuminating the whole of the 'light' compartment evenly (300-400 Lux). The dark zone was sheltered from the light by an opaque lid and a door at the opening between the two compartments. The mouse was placed in the centre of the dark compartment. The door was released at the start of the trial. Trial duration was 10 minutes, and two mice were tested in parallel. The time spent in each of the compartments, as well as the frequency of the transition between zones, was recorded. Significant differences between genotypes was identified using a Student's t-test (α=0.05). Mice that do not transition into the light zone at all during the duration of the assay are excluded due to the possibility that the apparent preference is due to a lack of exploration. One $Kptn^{-/-}$ mutant was excluded from the final analysis because it remained the dark zone the whole time.

Social recognition (SoR)

To test olfactory-mediated memory, the SoR assay was used based on previously published protocols[7,8]. Test female animals were habituated to the room (and red light) for half an hour in their home cages and were then habituated to the arenas for 10 minutes before the start of the testing. Stimuli were matched to test animals according to their weight, size, and gender. The familiar-unfamiliar stimuli pair were from different breeding colony, but of the same genetic background (129P2/OlaHsdWtsi + C57BL/6J), and counterbalanced for novelty. Mice were exposed to the same stimulus animals for four consecutive 1 min trials, with a 10 min inter-trial-interval. In trial 5 (1 min), a novel C57BL/6NTac mouse was used. For each trial, the same stimulus was used for 3 test animals to reduce the number of animals needing to be anaesthetised. On Day 2, after a 10 min habituation trial, mice were tested in a 2 min discrimination trial in which they were simultaneously exposed to two stimulus animals, a familiar mouse previously encountered on Day 1 (Trial 1 to 4) and a novel unfamiliar mouse. The time spent closely investigating the stimulus animals was recorded. Significant differences in investigation times were identified using two-way ANOVA with repeated measures for the trial/stimulus mouse (α=0.05). Post-hoc testing with Bonferroni correction was applied to identify specific significant differences, namely between trial 4 and trial 5. Stimulus animals were subject to non-terminal anaesthesia with ketamine/xylazine (intraperitoneal injection 100mg/10mg per kg of body weight) and were recovered with atipamezole (subcutaneous injection 1mg/kg or 0.025mg) in a home cage with a warm water bottle at the end of the experiment. The social recognition index (SRI) is defined as the difference in time spent investigating the novel versus familiar stimulus mouse as a fraction of total investigation time (novel mouse investigation time - familiar mouse investigation time / all investigation time). The inherent tendency of wildtype mice to investigate novel conspecifics leads to a higher SRI, with a maximum of 1 (i.e. **all** time spend investigating novel mouse) while a decrease in this

tendency (due to memory deficit, or other reasons) leads to an SRI of zero (**equal** time spent with both stimulus mice), and any tendency reversing wildtype behaviour (e.g. aversion to novel mouse, more time with familiar mouse) drives the SRI towards a minimum of -1. The social recognition indices were compared using an unpaired, two-sample, two-tailed Student's t-test (α=0.05) between genotypes, and one-sample two-tailed Student's t-tests (against population mean of zero) for within-genotype assessment. Mice were excluded if their overall investigation time was <10 seconds on trial 1 or if they did not investigate one of the stimuli during Day 2. One wildtype and four mutants were excluded due to the low investigation on trial 1 on Day 1 (final: *Kptn*$^{+/+}$ *n*=11, *Kptn*$^{-/-}$ *n*=8).

Pairwise discrimination

Pairwise visual discrimination (PD) assay was run using an automated touchscreen technology[9–11] (Bussey-Saksida chamber). The protocol used was adapted from previous studies and the Campden instruction manual for PD task (for mouse touch screen systems and ABET II)[10–12]. Because the assay is dependent on appetitive reward, the mice were food restricted to achieve a gradual reduction of 10-15% from their initial body weight, and this weight was maintained throughout training and testing. The weight of the mice was measured daily, and the food was adjusted accordingly. All the mice were progressed through the assay on an individual basis. Mice were tested in the same testing box each time throughout all the sessions. Once each mouse reached the criteria in the PD task phase they were no longer tested. In order to represent the percentage of correct trials over sessions in PD task, for those mice that completed the PD task earlier, the criteria mean (correct trial percentage over the last two days) was calculated, and this number was included into the overall mean for the subsequent sessions. Therefore, when the percentage of trials was plotted against sessions, each session had an equal number of mice, even though some mice finished the assay faster than others. The actual number of days taken to reach criteria was plotted separately (Student's two-tailed t-test,

α=0.05). There was a predefined 25 days cut-off point, after which if mice did not complete the criteria, they were excluded.

Barnes maze

The Barnes maze assay was run as described previously[13–15]. External spatial cues were placed around the walls of the room, to aid navigation. The mice were tested in a darkened room with overhead lights facing the Barnes maze tables (120 cm diameter with 20 x 5cm diameter holes), under maximum illumination. Three days before the first session, their cages were cleaned and mice were familiarised with the goal box, by placing one in the centre of the clean home cage. Mice were placed into the box and allowed to climb out of it. If the mouse did not leave the box after one minute, it was gently encouraged back out into the cage. The mice were tested sequentially and in the same order on every day, with two mice tested in parallel on two tables. Fifteen minutes before testing, mice were singly housed in new cages with bedding, food, a cardboard play tunnel, and a handful of old bedding from the home cage of the mouse. Mice were trained to find the goal box for two days (Training 1, four trials each day) and tested on Day 3 with a 24 h Probe trial. Animals were then retrained to a new location for 10 trials (Training 2); two trials on Day 3, four trials on Day 4 and another four trials on Day 5. On Day 8, mice were tested again with a 72 h Probe trial.

The time it took the mouse to find the goal box (primary latency), was recorded for each training trial. For the Probe trials, the time spent in the areas around each of the holes (10cm diameter area) and the average distance away from each of the holes was recorded. The time around each hole was calculated as a percentage of the total time spent around the holes (i.e. the sum of all the intervals in proximity to the holes). Significant differences between genotypes were identified using two-way ANOVA with genotype and hole/training day as factors (α=0.05).

MRI (Magnetic Resonance Imaging)

For MRI analysis, skulls were collected after transcardial perfusion, and stored in formalin solution at 4C. MRI imaging and analysis were performed, using previously published methodology[16]. For female mice, we used *n*=8 for each genotype, For male mice, we used 6 wildtype and 7 homozygous mutant animals. Brains were scanned using a Bruker PharmaScan 47/16 system at 4.7T with a manufacturer-provided birdcage transmit-receive coil. The images were registered into the same stereotactic space and segmented into images of four tissue types: grey matter, white matter, cerebrospinal fluid and 'other'. This was followed by voxel-based quantification of brain volume[17]. To control the type I error rate due to multiple comparisons, an adjusted p-value was used for a false discovery rate at $p < 0.05$.

Microcomputed X-ray tomography

Skulls of male mice (*n*=5 per genotype) were collected after transcardial perfusion and stored in formalin solution at 4C until they were ready to be scanned. High-resolution images were acquired using the 1172 Bruker microCT scanner. The scanning parameters were set to: 2000 x 1332 object position, pixel size 9.89, Medium camera size. Prior to acquisition, we ensured the average intensity value (av=xx) was in the acceptable range of 40-60% (no more than 70%) and the 'min' value was ≥10%. DICOM files generated by the Bruker software were loaded into 3DimViewer (V3.0, http://www.3dim-laboratory.cz) for surface model creation using default thresholding (1482/7000) and saved as an .STL file. The STL files were loaded individually into Meshlab software[18] (V2016.12, www.meshlab.net), and individual landmark locations[19] were recorded (Supplemental Fig. 3) . Inter-landmark distances were calculated in excel. Significant differences in mean linear distances were identified using a Student's t-test (α=0.05). Visualisation, 3d rendering and resectioning was performed in Meshmixer V3.5.474 (Autodesk, Inc., www.meshmixer.com).

Histomorphometric analysis

For the histomorphological analysis, adult mice aged 16 weeks (*n*=8 per genotype and sex) were transcardially perfused, the brain was removed from the skull and post-fixed in formalin for 48 hours, after which it was kept in PBS. For the P0 brains, male mice were culled at birth, brains were removed from skull and post-fixed for 12 days before being transferred into PBS. The morphometric analyses were carried out using a previously published high-throughput method[20,21], examining the same three coronal brain regions (section 1: Bregma +0.98mm; section 2: Bregma -1.34mm; and section 3: Bregma -5.80mm) in all mice. Mice were separately analysed according to their sex. Sections were scanned using Slide scanner (Hamamatsu, NanoZoomer 2.0HT, C9600 series) and accessories (racks and NanoZoomer digital pathology, version 2.5.64 software) at 20x magnification and analysed using ImageJ (78 measurements for P20 and 16-week animals, 53 measurements for P0 animals, as listed in Supplemental File 1).

Immunohistochemistry

Adult mice were euthanized by CO2 asphyxiation followed by thoracotomy. All animals were perfused with ice-cold saline and 4% PFA. Brains were dissected out and post-fixed in 4% PFA for 24 h. After fixation, brains were embedded in paraffin wax, sliced on a microtome at 8 μm, mounted on slides, and hot air dried overnight. Following drying, brains were deparaffinized, subjected to antigen retrieval via citrate buffer, and washed in distilled water. Washed slides were blocked for 1 h at room temperature (RT) in blocking solution containing 0.3% tween PBS with 5% normal goat serum and incubated in primary antibodies overnight in blocking solution. Primary antibodies used were anti-phospho-RPS6 (ser240/244; #5364, Cell Signaling Technology; 1:1000; rabbit polyclonal) and RPS6 (#F52532, NSJ Bioreagents; 1:100, mouse monoclonal). After incubation with primary antibodies, slides were washed in PBS and placed in blocking solution with secondary antibodies for 2 h at RT. Secondary antibodies were conjugated with Alexa 594 (Invitrogen; 1:1000; goat anti-rabbit) and 647

(Invitrogen; 1:1000; goat anti-rabbit) fluorophores, respectively. After two hours, slides were washed in PBS and distilled water and finally coverslipped with Vectashield media containing DAPI. Slides were then imaged on a Nikon spinning disk confocal microscope and digital micrographs were created in Imaris software (Oxford Instruments).

Tissue lysis and immunoblotting

For protein extracts used in immunoblotting, PBS perfused tissue was collected as described above, snap frozen in liquid nitrogen, and stored in -80C until lysis. Tissue was lysed in 1x lysis solution (protease + phosphatase inhibitor cocktail in TPER) in the Qiagen TissueLyser LT with sterile steel beads and operated at 30Hz for 2 minutes. Samples were spun at 4C for 15 minutes at 15000 RPM and the supernatant was kept on ice. Bio-Rad DC protein assay kit was used for protein quantifications, following the manufacturer′s protocol. Samples were boiled for 5 minutes and then subjected to XCell4 SureLock Midi-Cell electrophoresis. 20uL of 30ug of protein was run per well. Membranes were blocked for an hour in 5% BSA (in PBS with 0.1% Tween). Primary antibodies were incubated overnight at 4C, followed by 1 h incubation in horseradish peroxidase–conjugated secondary antibody (anti-rabbit or anti-mouse, #7074 and #7076, Cell Signaling Technology) diluted in PBS-T 5% milk at room temperature. Images were acquired with a digital imaging system (ImageQuant LAS 4000, GE Healthcare) and analysed using ImageJ software. Primary antibodies: anti-RPS6 (clone 54D2, #2317, Cell Signaling Technology), Ser240/244-specific anti-phospho-RPS6 (#2215, Cell Signaling Technology), and anti-GAPDH (ab9485, Abcam).

Mutagenesis and differentiation of KPTN iPSC models

iPSC experiments were all performed in an isogenic background: Kolf2C1_WT (HPSI0114i-kolf_2), feeder-free hiPSC, male, derived from skin tissue using Cytotune 1 reprogramming[22]. In order to create knockout iPSC lines, a guide RNA (gRNA) was selected to target coding

exon 8 (guide location: GRCh38:19:47479882-47479904) of the human KPTN gene. The synthetic gRNA along with Cas9 protein was delivered into wild-type Kolf2C1 cells[22] as a pre-complexed ribonucleoprotein via electroporation. The addition of a short single-stranded oligodeoxynucleotide of non-complementary sequence was also added to improve delivery. After a period of recovery, the cells were subcloned and submitted for genotyping by Miseq sequencing. Clones were screened for the presence of frameshift-causing indels and then expanded for banking. Clones identified as unedited in the genotyping analysis were selected as unedited controls, to control for electroporation, selection, and passaging effects. After banking, and subsequent to all differentiation experiments, all cell lines were sent for a second round of confirmatory genotyping. This work was performed by the Gene Editing facility at the Wellcome Sanger Institute.

Differentiation of iPSCs to cortical neural precursor cells

KPTN mutant heterozygous, homozygous edited and wild-type control IPSC lines were cultured in feeder-free conditions as per published protocols[23,24]. Dual-SMAD inhibition (SB431542, #ab120163, Abcam and LDN193189, #2092-5, Cambridge Bioscience) was used in a 10-day neural induction in the presence of XAV939 (#3748, Tocris) to promote regional forebrain identity[23]. After passaging at day 10, cells were replated for immunocytochemical quality control, and then banked at day 14 for RNA extraction and RNA-Seq analysis. All experiments were confirmed to have high PAX6/NESTIN positivity (>90% of cells at day 14). Antibodies for NPC quality assessment included anti-NESTIN (1:100, rabbit monoclonal, clone #SP103, ab105389Abcam), anti-PAX6 (1:200, rabbit monoclonal, clone #D3A9V), 60433S, Cell Signaling Technology), and anti-OCT-3/4 (1:100, mouse monoclonal, clone #C-10, Santa Cruz Biotechnology) . Secondary antibodies used were Donkey anti-Rabbit and anti-mouse Alexa Fluors(#A-21206 and #A-31571, Invitrogen). All wildtype, heterozygous, and

homozygous clones (n=2,2,1 respectively) were differentiated in duplicate, providing 2-4 separate biological replicates per genotype (n=4,4,2 replicates respectively).

iPSC RNA extraction and RNA sequencing

RNA extraction and sequencing of iPSC derived materials was performed using manufacturer's protocols for the RNeasy QIAcube kit on a QIAcube automated system (Qiagen). RNA sequencing libraries were prepared using established protocols: library construction (poly(A) pulldown, fragmentation, 1st and 2nd strand synthesis, end prep and ligation) was performed using the NEB Ultra II RNA custom kit (New England Biolabs) on an Agilent Bravo automated system. Indexed multiplexed sequencing was performed on the Novaseq 6000 system (S4 flow cell, Xp workflow; Illumina), collecting approximately 30 million paired-end reads per sample with 100 base read length. The bulk RNA sequencing data were de-multiplexed into separate CRAM files for each library in a lane. Adapters that had been hard-clipped prior to alignment were reinserted as soft-clipped post alignment, and duplicated fragments were marked in the CRAM files. The data pre-processing, including sequences QC, and STAR alignments were made with a custom Nextflow pipeline, which is publicly available at https://github.com/wtsi-hgi/nextflow-pipelines/blob/rna_seq_5607/pipelines/rna_seq.nf, including the specific aligner parameters. We assessed the sequence data quality using FastQC v0.11.8. Reads were aligned to the GRCh38 human reference genome (Ensembl GTF annotation v91). We used STAR[25] version STAR_2.6.1d with the --twopassMode Basic parameter. The STAR index was built against GRCh38 Ensembl GTF v91 using the option -sjdbOverhang 75. We then used featureCounts version 1.6.4[26] to obtain a readcount matrix. Genes with no count or only a single count across all samples were filtered out. The counts were normalised using DESeq2’s median of ratios method[27]. Differential gene expression was analysed using the DESeq2 package[27] with SVA correction[28]. An adjusted p-value threshold of 0.05 was selected to identify significant differences between wild-type and mutant samples.

Mouse RNA extraction and RNA sequencing

For wild-type and homozygous *Kptn* mouse samples, we used *n*=5-6 per genotype and tissue as follows (wildtype/mutant): E18 brain, *n*=6/6; P21 hippocampus, *n*=5/6; P21 cortex, *n*=5/6; adult cerebellum, *n*=5/6; adult hippocampus, *n*=6/6; adult cortex, *n*=5/5. Fresh frozen mouse tissues were homogenised in buffer RLT plus (Qiagen) with β-mercaptoethanol (Sigma, M3148; 10µl/ml) using Qiagen TissueLyser LT, with sterile RNAse-ZAP treated steel beads and operated at 50Hz for 2 minutes. Samples were pre-treated on gDNA eliminator columns and then extracted on RNeasy Plus columns as per manufacturer's protocol (Qiagen), and were immediately snap frozen on dry ice and stored at -80C. An aliquot of each sample was quantified using 2100 Bioanalyzer (Agilent Technologies). Library preparation was performed by Wellcome Sanger Institute DNA Pipelines, as described above for iPSCs. Samples were sequenced using 75 bp paired-end sequencing reads (reverse stranded) on a Illumina-HTP HiSeq 4000 system. The bulk RNA sequencing data were de-multiplexed into separate CRAM files for each library in a lane. Adapters that had been hard-clipped prior to alignment were reinserted as soft-clipped post alignment, and duplicated fragments were marked in the CRAM files. The data pre-processing, including sequences QC, and STAR alignments were made with a Nextflow pipeline, which is publicly available at https://github.com/wtsi-hgi/nextflow-pipelines/blob/rna_seq_mouse/pipelines/rna_seq.nf, including the specific aligner parameters. We assessed the sequence data quality using FastQC v0.11.8. Reads were aligned to the GRCm38 mouse reference genome (Mus_musculus.GRCm38.dna.primary_assembly.fa, Ensembl GTF annotation v99). We used STAR version 2.7.3a[25] with the --twopassMode Basic parameter. The STAR index was built against Mus_musculus GRCm38 v99 Ensembl GTF using the option -sjdbOverhang 75. We then used featureCounts version 2.0.0[26] to obtain a readcount matrix. The count data was used as input for differential gene expression analysis

using DESeq2 package[27] with SVA correction[28]. The default DESeq2 cut-off of BH-adjusted p-value<0.1 was used for the mouse RNA-Seq analyses.

Psychometric testing of *KPTN*-related disorder probands

Six Amish individuals between the ages of 11 and 29 with KRD (3 female, 3 male) were psychometrically assessed using the Wechsler Intelligence Scale for Children 4th Edition (WISC-IV) (6-16 years) or the Wechsler Adult Intelligence Scale 4th Edition (WAIS-IV) (over 16 years), which assesses cognitive performance in four domains including verbal comprehension (VCI), perceptual reasoning (PRI), processing speed (PSI) and working memory (WMI) indices, and can be combined to generate a full-scale intelligence quotient (FSIQ) score. Immediate memory recall was assessed using the story memory test and list learning test from the developmental NEuroPSYchological Assessment- 2nd Edition (NEPSY-II) (5-16 years) or the Repeatable Battery for Assessment of Neurological Status- 2nd Edition (RBANS-Update) (over 12 years). All researchers collecting assessment data were trained and supervised by a Clinical Psychologist. The WISC-IV and WAIS-IV tests have normalised reference scores, with mean of 100 and an SD of 15, although to allow comparison standardised scores were converted to Z-scores. The NEPSY-II and RBANS-Update tests generate scaled scores, with mean of 10 and an SD of 3, which were also converted to Z-scores for direct comparison. As none of these tests have been validated within the Amish population and due to possible cultural differences that could affect test scores, this necessitated data collection from a reference control group. Six age-matched Amish control individuals (6 female) who were unaffected siblings or relatives of the six affected individuals, underwent the same psychometric tests, to serve as controls for this analysis.

Statistical methods

All statistical tests are listed in the relevant sections above.

Supplemental references

1. Skarnes WC, Rosen B, West AP, et al. A conditional knockout resource for the genome-wide study of mouse gene function. *Nature*. 2011;474(7351):337-342. doi:10.1038/nature10163
2. Testa G, Schaft J, van der Hoeven F, et al. A reliable lacZ expression reporter cassette for multipurpose, knockout-first alleles. *Genesis*. 2004;38(3):151-158. doi:10.1002/gene.20012
3. White JK, Gerdin AK, Karp NA, et al. Genome-wide generation and systematic phenotyping of knockout mice reveals new roles for many genes. *Cell*. 2013;154(2):452-464. doi:10.1016/j.cell.2013.06.022
4. Seibenhener ML, Wooten MC. Use of the Open Field Maze to measure locomotor and anxiety-like behavior in mice. *J Vis Exp*. 2015;(96):e52434. doi:10.3791/52434
5. Hascoët M, Bourin M. The mouse light-dark box test. *Neuromethods*. 2009;42:197-223. doi:10.1007/978-1-60761-303-9-11
6. Crawley J, Goodwin FK. Preliminary report of a simple animal behavior model for the anxiolytic effects of benzodiazepines. *Pharmacol Biochem Behav*. 1980;13(2):167-170.
7. Dias C, Estruch SB, Graham SA, et al. BCL11A Haploinsufficiency Causes an Intellectual Disability Syndrome and Dysregulates Transcription. *The American Journal of Human Genetics*. 2016;99(2):253-274. doi:10.1016/j.ajhg.2016.05.030
8. Sánchez-Andrade G, James BM, Kendrick KM. Neural Encoding of Olfactory Recognition Memory. *Journal of Reproduction and Development*. 2005;51(5).
9. Bubser M, Bridges TM, Dencker D, et al. Selective Activation of M $_4$ Muscarinic Acetylcholine Receptors Reverses MK-801-Induced Behavioral Impairments and Enhances Associative Learning in Rodents. *ACS Chem Neurosci*. 2014;5(10):920-942. doi:10.1021/cn500128b
10. Horner AE, Heath CJ, Hvoslef-Eide M, et al. The touchscreen operant platform for testing learning and memory in rats and mice. *Nat Protoc*. 2013;8(10):1961-1984. doi:10.1038/nprot.2013.122
11. Morton AJ, Skillings E, Bussey TJ, Saksida LM. Measuring cognitive deficits in disabled mice using an automated interactive touchscreen system. *Nat Methods*. 2006;3(10):767. doi:10.1038/nmeth1006-767
12. Brigman JL, Daut RA, Wright T, et al. GluN2B in corticostriatal circuits governs choice learning and choice shifting. *Nat Neurosci*. 2013;16(8):1101-1110. doi:10.1038/nn.3457
13. Harrison FE, Reiserer RS, Tomarken AJ, McDonald MP. Spatial and nonspatial escape strategies in the Barnes maze. *Learn Mem*. 2006;13(6):809-819. doi:10.1101/lm.334306
14. Harrison FE, Hosseini AH, McDonald MP. Endogenous anxiety and stress responses in water maze and Barnes maze spatial memory tasks. *Behavioural Brain Research*. 2009;198(1):247-251. doi:10.1016/j.bbr.2008.10.015
15. Koopmans G, Blokland A, van Nieuwenhuijzen P, Prickaerts J. Assessment of spatial learning abilities of mice in a new circular maze. *Physiol Behav*. 2003;79(4-5):683-693. doi:10.1016/s0031-9384(03)00171-9
16. Sawiak SJ, Wood NI, Williams GB, Morton AJ, Carpenter TA. Voxel-based morphometry with templates and validation in a mouse model of Huntington's disease. *Magn Reson Imaging*. 2013;31(9):1522-1531. doi:10.1016/j.mri.2013.06.001
17. Ashburner J, Friston KJ. Voxel-based morphometry--the methods. *Neuroimage*. 2000;11(6 Pt 1):805-821. doi:10.1006/nimg.2000.0582

18. Cignoni P, Callieri M, Corsini M, Dellepiane M, Ganovelli F, Ranzuglia G. MeshLab: An open-source mesh processing tool. *6th Eurographics Italian Chapter Conference 2008 - Proceedings*. Published online 2008:129-136.
19. de Carlos F, Alvarez-Suarez A, Costilla S, Noval I, Vega JA, Cobo J. 3D-μCT Cephalometric Measurements in Mice. In: *Computed Tomography - Special Applications*. InTech; 2011. doi:10.5772/24234
20. Mikhaleva A, Kannan M, Wagner C, Yalcin B. Histomorphological Phenotyping of the Adult Mouse Brain. *Curr Protoc Mouse Biol*. 2016;6(3):307-332. doi:10.1002/cpmo.12
21. Collins SC, Mikhaleva A, Vrcelj K, et al. Large-scale neuroanatomical study uncovers 198 gene associations in mouse brain morphogenesis. *Nat Commun*. 2019;10(1):3465. doi:10.1038/s41467-019-11431-2
22. Kilpinen H, Goncalves A, Leha A, et al. Common genetic variation drives molecular heterogeneity in human iPSCs. *Nature*. 2017;546(7658):370-375. doi:10.1038/nature22403
23. Qi Y, Zhang XJ, Renier N, et al. Combined small-molecule inhibition accelerates the derivation of functional cortical neurons from human pluripotent stem cells. *Nat Biotechnol*. 2017;35(2). doi:10.1038/nbt.3777
24. Neaverson A, Andersson MHL, Arshad OA, et al. Differentiation of human induced pluripotent stem cells into cortical neural stem cells. *bioRxiv*. Published online January 1, 2022:2022.08.19.504404. doi:10.1101/2022.08.19.504404
25. Dobin A, Davis CA, Schlesinger F, et al. STAR: ultrafast universal RNA-seq aligner. *Bioinformatics*. 2013;29(1):15-21. doi:10.1093/bioinformatics/bts635
26. Liao Y, Smyth GK, Shi W. featureCounts: an efficient general purpose program for assigning sequence reads to genomic features. *Bioinformatics*. 2014;30(7):923-930. doi:10.1093/bioinformatics/btt656
27. Love MI, Huber W, Anders S. Moderated estimation of fold change and dispersion for RNA-seq data with DESeq2. *Genome Biol*. 2014;15(12):550. doi:10.1186/s13059-014-0550-8
28. Leek JT, Storey JD. Capturing heterogeneity in gene expression studies by surrogate variable analysis. *PLoS Genet*. 2007;3(9):1724-1735. doi:10.1371/journal.pgen.0030161

Author contributions

D.W.L., A.C., E.L.B., M.O.L., and S.S.G. conceived of the original project; M.O.L., G.S.A., D.W.L., M.E.H., and S.S.G. designed mouse experiments; D.W.L., M.E.H., and S.S.G. supervised the project; E.L.Cambridge, L.T., and M.S. performed initial mouse phenotyping; C.J.L. supervised initial mouse phenotyping; M.O.L, D.W.L., G.S.A., E.L.Coomber, M.S., C.R., and S.S.G carried out mouse behavioural phenotyping; L.E.R., E.L.B., and A.C. supervised clinical data collection, and psychometric testing; J.T. and L.J. performed psychometric testing and analysis; C.B., I.E., J.C.H., S.G.K., E.N., A.Y.S., E.M.S., H.S., J.S., T.T., C.V.R.A., P.V., M.W., and O.W. provided clinical data; L.E.R., and E.L.B. collated clinical data; M.O.L., G.S.A., and H.I. performed sample preparation, western blotting, and qPCR; S.C.C. performed mouse histological processing and analysis; B.Y. supervised mouse histological brain analysis; M.O.L. carried out μCT scans; S.S.G. analysed μCT data; P.H.I.II performed mouse immunochemistry; P.B.C. supervised mouse immunochemistry, and contributed to manuscript preparation; S.J.S. carried out MRI scanning and analysis; A.D., A.H., and M.P. supervised iPSC work; M.H.L.A, A.N., and E.R. carried out iPSC differentiations; M.O.L., G.S.A., M.S., and S.S.G analysed mouse behavioural data; G.S.A., O.A., and S.S.G. analysed RNA-Seq results; M.O.L., G.S.A., L.E.R., E.L.B., and S.S.G. wrote the manuscript, and assembled figures.

A

```
Exon 8(NM_007059.4): AGGTTCTGCAGATGTGGTCGGTCCTGCAGGACGGTCCCATCTCCCGAGTGATTGTGTTCAGCCTCTCGGCCGCCAAGG
c.714_731dup:        AGGTTCTGCAGATGTGGTCGGTCCTGCAGGACGGTCCCATCTCCCGAGTGATTGTGTTCAGCCTCTCGGCCGCCAAGG
c.776C>A:            AGGTTCTGCAGATGTGGTCGGTCCTGCAGGACGGTCCCATCTCCCGAGTGATTGTGTTCAGCCTCTAGGCCGCCAAGG
KPTN -/- allele 1:   AGGT-------------------------GACGGTCCC-------GAGTGATTGTGTTCAGCCTCTCGGCCGCCAAGG
KPTN -/- allele 2:   AGGTTCTGCAGATGTGGTCGGTCCTGCAGGACGGTCCC-------GAGTGATTGTGTTCAGCCTCTCGGCCGCCAAGG
KPTN +/- allele 1:   AGGTTCTGCAGATGTGGTCGGTCC--------------------CGAGTGATTGTGTTCAGCCTCTCGGCCGCCAAGG
```

B **C**

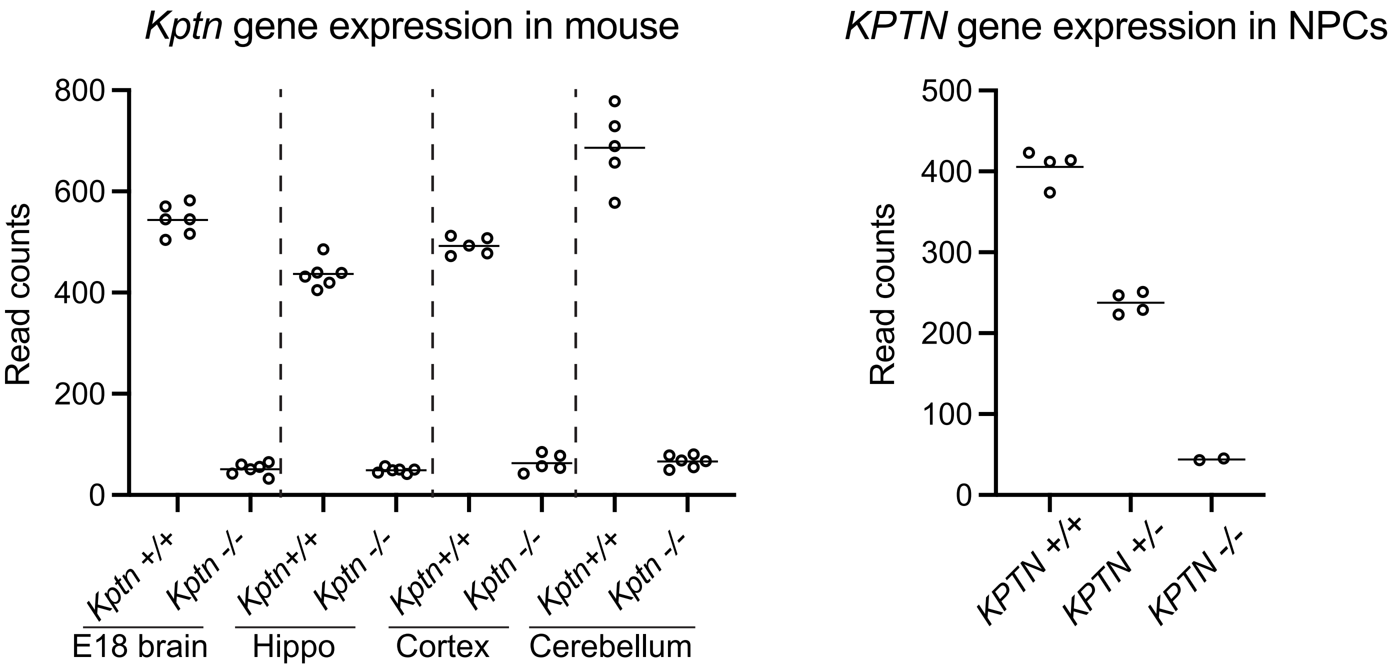

SUPPLEMENTARY FIGURE 1 Kptn gene expression in mouse and human loss-of-function models. **A** Alignment of Exon 8 *KPTN* alleles reported in Baple et al. 2014 (c.714_731dup and c.776C>A), and in hIPSCs used in this study (allele 1: c717_c741del + c751_757del, allele 2: c751_757del, allele 3: c734_753del). All coordinates refer to MANE select transcript (NM_007059.4). Duplicated regions are highlighted in red, missense changes in red font, and deletions represented with dashes. **B** Normalised RNA-Seq read counts for Kptn in embryonic (E18 brain) and adult (Hippocampus, Cortex, Cerebellum) samples of *Kptn*$^{-/-}$ mice and *Kptn*$^{+/+}$ controls. **C** Normalised RNA-Seq read counts for *KPTN* in differentiated neural precursor cells of *KPTN*$^{+/+}$, *KPTN*$^{+/-}$, and *KPTN*$^{-/-}$ genotypes.

Supplementary Figure 2

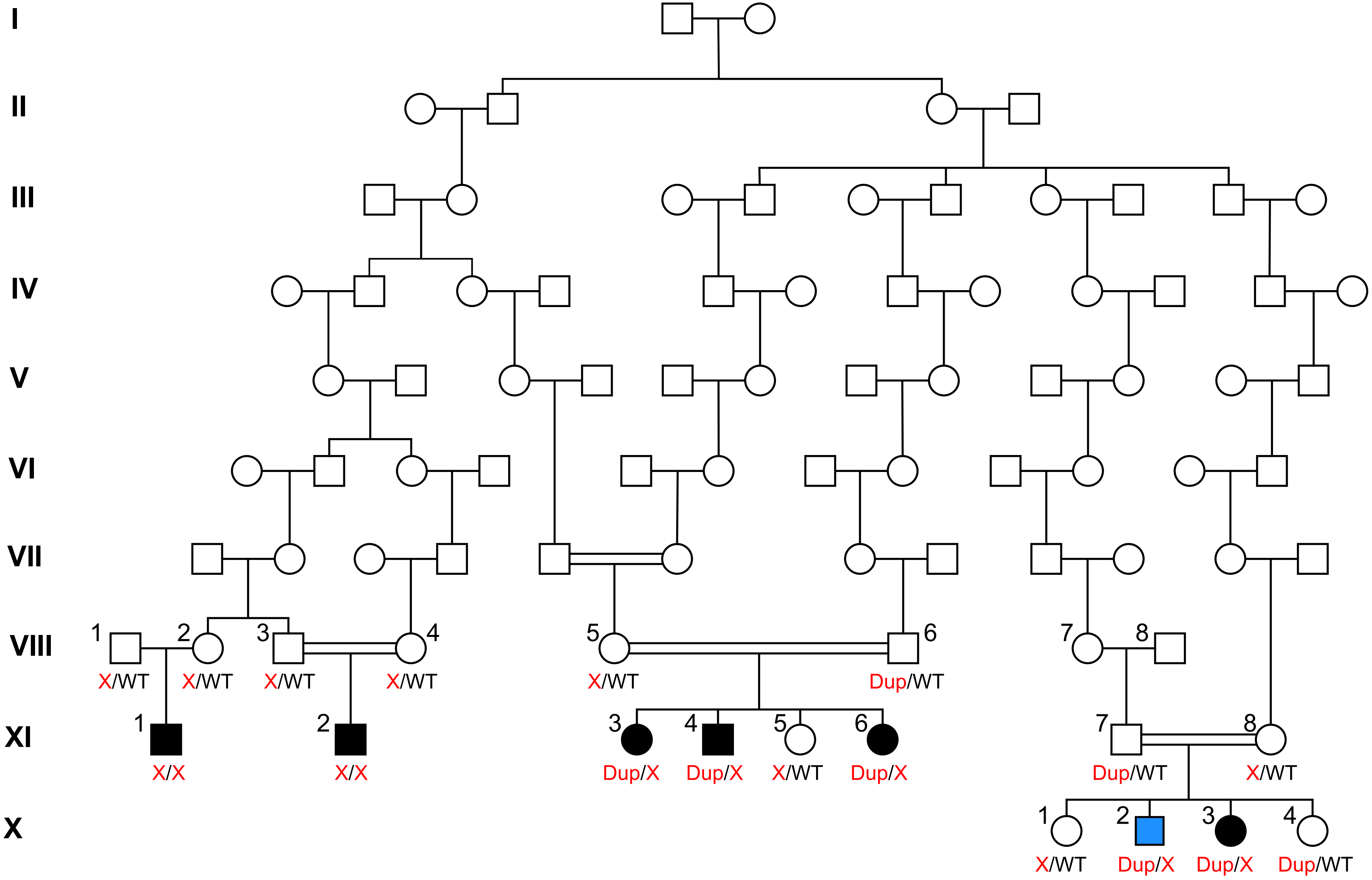

SUPPLEMENTARY FIGURE 2 Pedigree of an extended interrelated Amish family. Relationship between seven individuals with KRD who underwent psychometric testing (shaded symbols). Affected individual X:2 (blue shaded symbol); psychometric testing was attempted but was not possible due to the severity of the intellectual impairment. Genotype is shown in red under each individual (X, p.(Ser259*) allele; Dup, p.(Met241_Gln246dup) allele; WT, wild-type allele).

Supplementary Figure 3

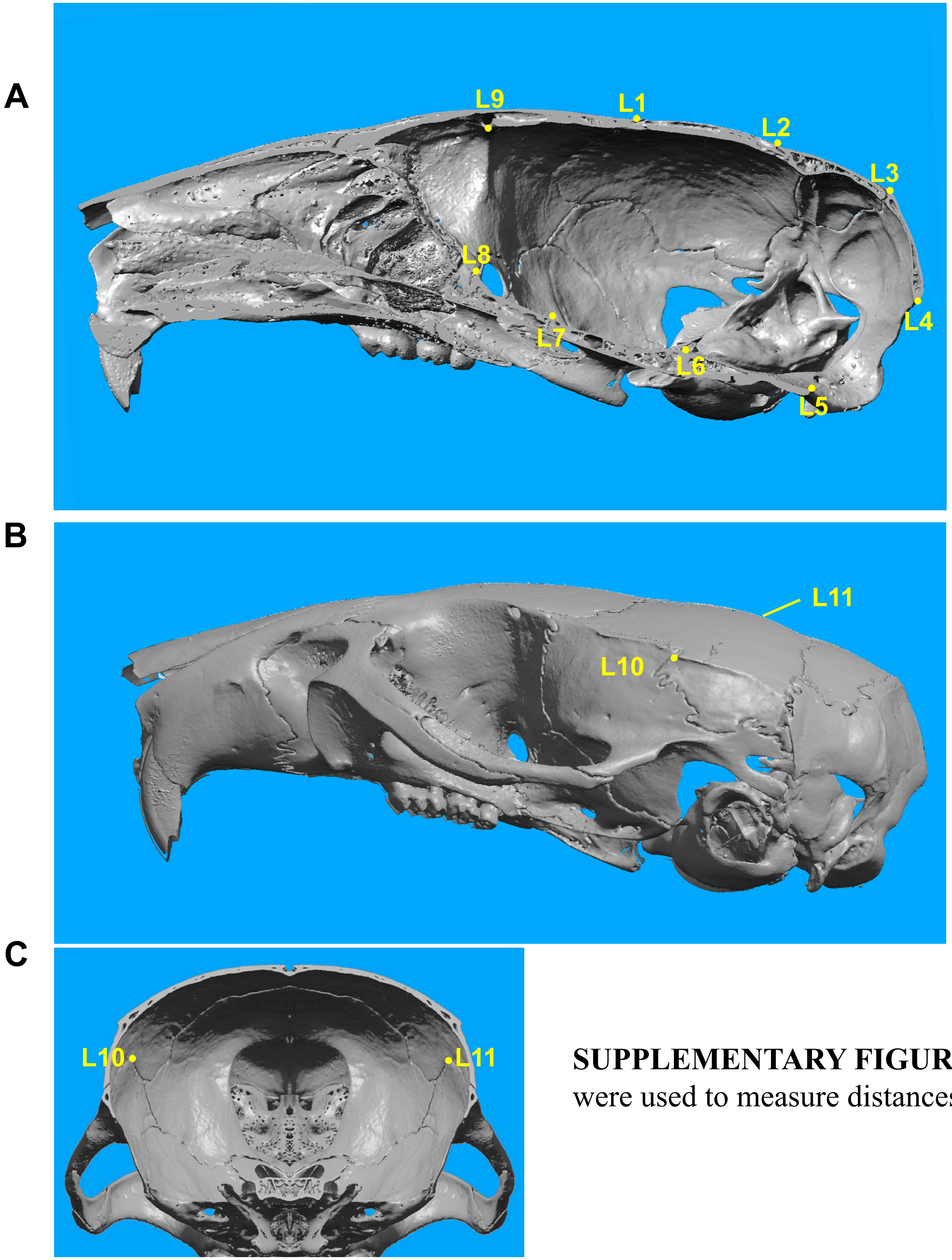

L1	Bregma
L2	Lambda
L3	Intersection of interparietal bones with squamous portion of occipital bone at midline
L4	Opisthion, midsagittal point on the posterior margin of the foramen magnum
L5	Caudal most point of basi-occipital bone at mid-sagittal plane
L6	Dorsal-most point of spheno-occipital synchondrosis at mid-sagittal plane
L7	Dorsal-most point of inter-sphenoid synchondrosis at mid-sagittal plane
L8	Rostral end of pre-sphenoid bone at mid-sagittal plane
L9	Caudal-most point of the roof of the olfactory fossa
L10	Intersection of the saquamosal suture with temporal crest, Left
L11	Intersection of the saquamosal suture with temporal crest, Right

SUPPLEMENTARY FIGURE 3 Landmarks used for microcomputed tomography analysis. L1-L11 landmarks were used to measure distances in adult mouse skulls of KRD models, as computed in Supplementary Table 1.

Supplementary Figure 4

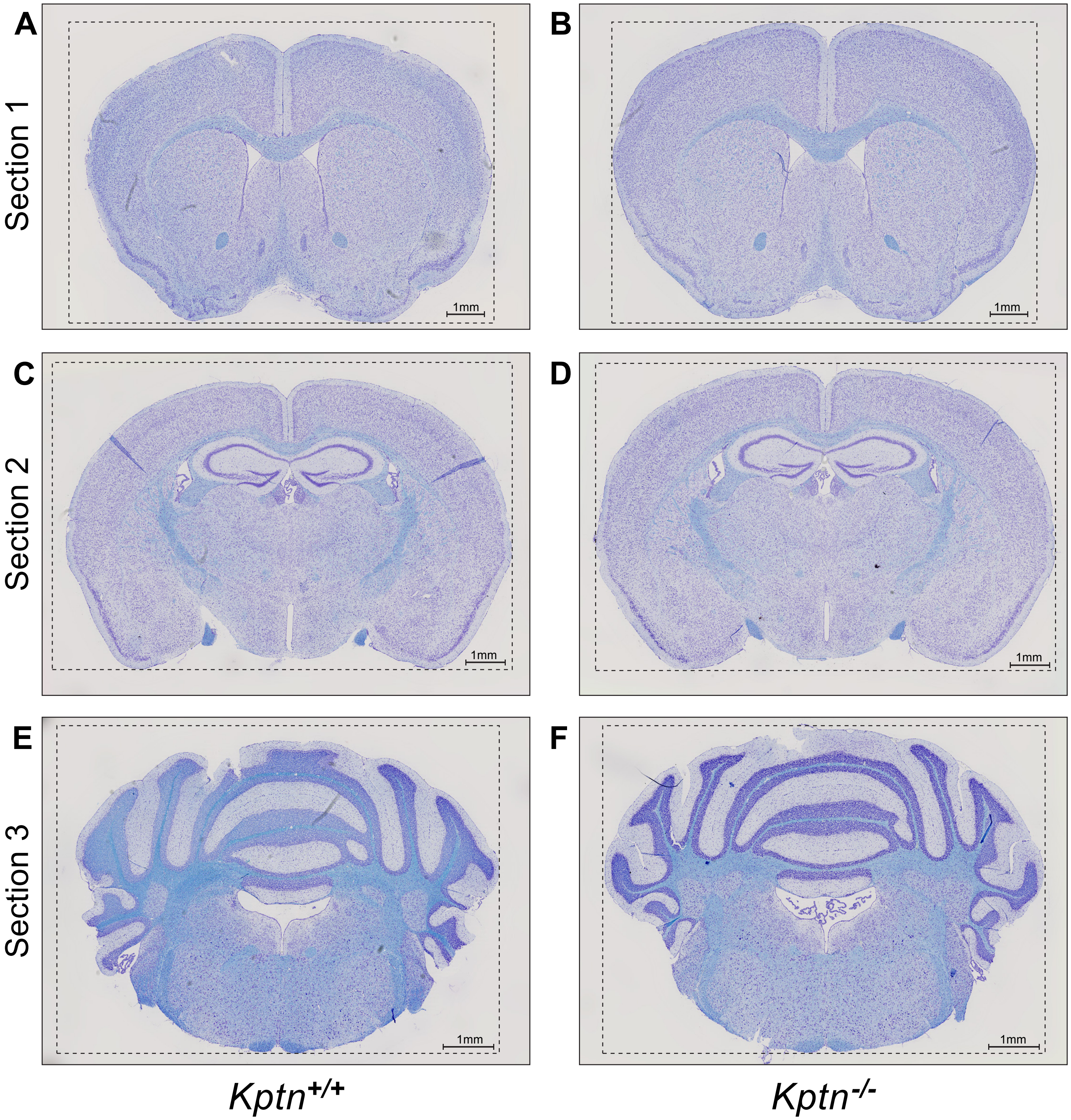

SUPPLEMENTARY FIGURE 4 Histological sections of mouse brains. Representative sagittal sections of the 3 regions used for neuroanatomical quantification, at **A,B** section 1 (Bregma +0.98mm), **C,D** section 2 (Bregma -1.34mm), and **E,F** section 3 (Bregma -5.80mm). Equally sized black dashed boxes denote the size of *Kptn*$^{-/-}$ samples for each region, which are visibly larger than their wildtype *Kptn*$^{+/+}$ controls in **A,C,E**. All scale bars are 1mm, and scale is matched per region.

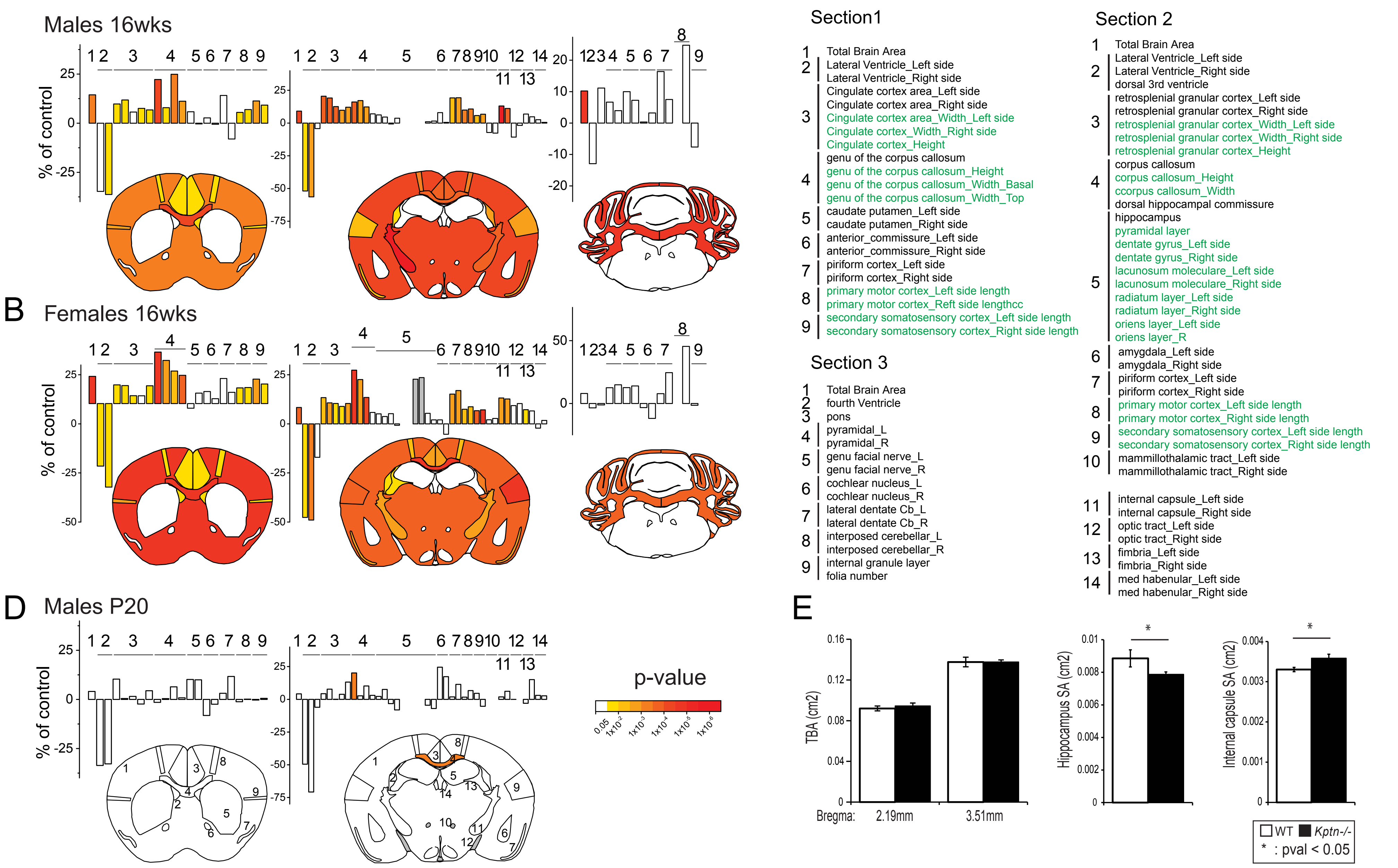

SUPPLEMENTARY FIGURE 5 Neuroanatomical assessment of the mouse KRD model. **A** Neuroanatomical findings in adult male Kptn mice (n=8 *Kptn*$^{-/-}$ versus 8 *Kptn*$^{+/+}$ controls). Below, a representative heatmap of the p-values for the three studied sections (Bregma +0.98mm, Bregma -1.34mm and Bregma -5.80mm). Above, histograms of the percentage change of *Kptn*$^{-/-}$ relative to *Kptn*$^{+/+}$ controls (100%). **B** Neuroanatomical findings in adult female Kptn mice (n=8 *Kptn*$^{-/-}$ versus 8 *Kptn*$^{+/+}$ controls), as described for males in **A**. In the legends in **C**, green text indicates length measurements and black text denotes area measurements. **D** Neuroanatomical findings in three week old (P20) male Kptn mice (n=6 *Kptn*$^{-/-}$, 6 *Kptn*$^{+/+}$ controls), as described for adults in **A**, with legend in **C. E** At Birth (P0), measurements of surface area for total brain area (tba), hippocampus, and internal capsule of *Kptn*$^{-/-}$ mice (n=9) compared to *Kptn*$^{+/+}$ controls (n=8). (See Supplementary File 1 for details). All p-values are from 2-sample two-tailed Student's t-tests.

Supplementary Figure 5

Supplementary Figure 6

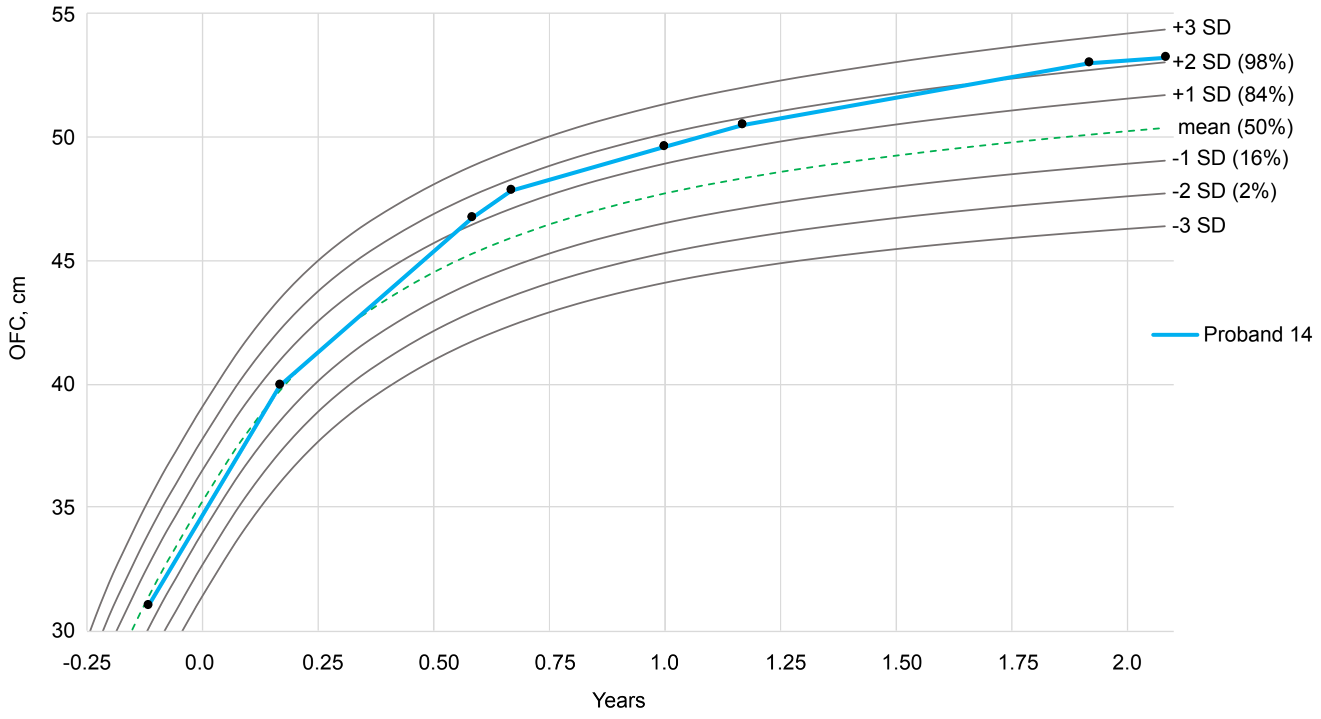

SUPPLEMENTARY FIGURE 6 Progressive macrocephaly in a representative KRD proband. Occipitofrontal circumference in centimetres for a male proband (Proband 14, Supplementary File 2) with KRD. OFC increased from below the 50th centile (mean) at birth to over two standard deviations (SD) above the mean by the age of two years (blue line). Centiles given in brackets.

Supplementary figure 7

A

Connectivity Map overall scores across all cell lines

Rank	Score	ID	Compound	Description	Target
1	-96.28	BRD-K68174511	torin-2	mTOR inhibitor	MTOR
2	-91.63	BRD-A99449986	MT-21	Caspase activator	CYCS, SLC25A4
3	-84.80	BRD-K37798499	etoposide	Topoisomerase inhibitor	TOP2A, CYP2E1, CYP3A5, TOP2B
4	-82.27	BRD-A25687296	emetine	Protein synthesis inhibitor	RPS2
5	-81.30	BRD-K36395411	SB-206553	Serotonic receptor antagonist	HTR2B, HTR2C, HTR1A, HTR2A
6	-80.70	BRD-K02130563	panobinostat	HDAC inhibitor	HDAC1, HDAC2, HDAC3, HDAC4, HDAC6, HDAC7, HDAC8, HDAC9

B

HCC515 cell line

Score	Rank	Compound	Description
-99.56	1	torin-2	mTOR inhibitor
-99.04	3	QL-X-138	mTOR inhibitor
-95.89	12	torin-1	mTOR inhibitor
-93.44	30	sirolimus	mTOR inhibitor
-93.23	32	WYE-354	mTOR inhibitor

SUPPLEMENTARY FIGURE 7 Connectivity Map search identifies mTOR inhibitors as the most likely effective treatment for KRD. Top negatively-ranked compounds identified by querying the Connectivity Map database with a differentially expressed gene set from *KPTN*$^{-/-}$ NPCs. **A** Torin-2 is identified as the strongest inversely-correlated compound across all cell types. **B** Query results of the Connectivity Map specifically in HCC515 cells identifies five known mTOR inhibitors among the top 32 negatively ranked compounds.

Supplementary Figure 8

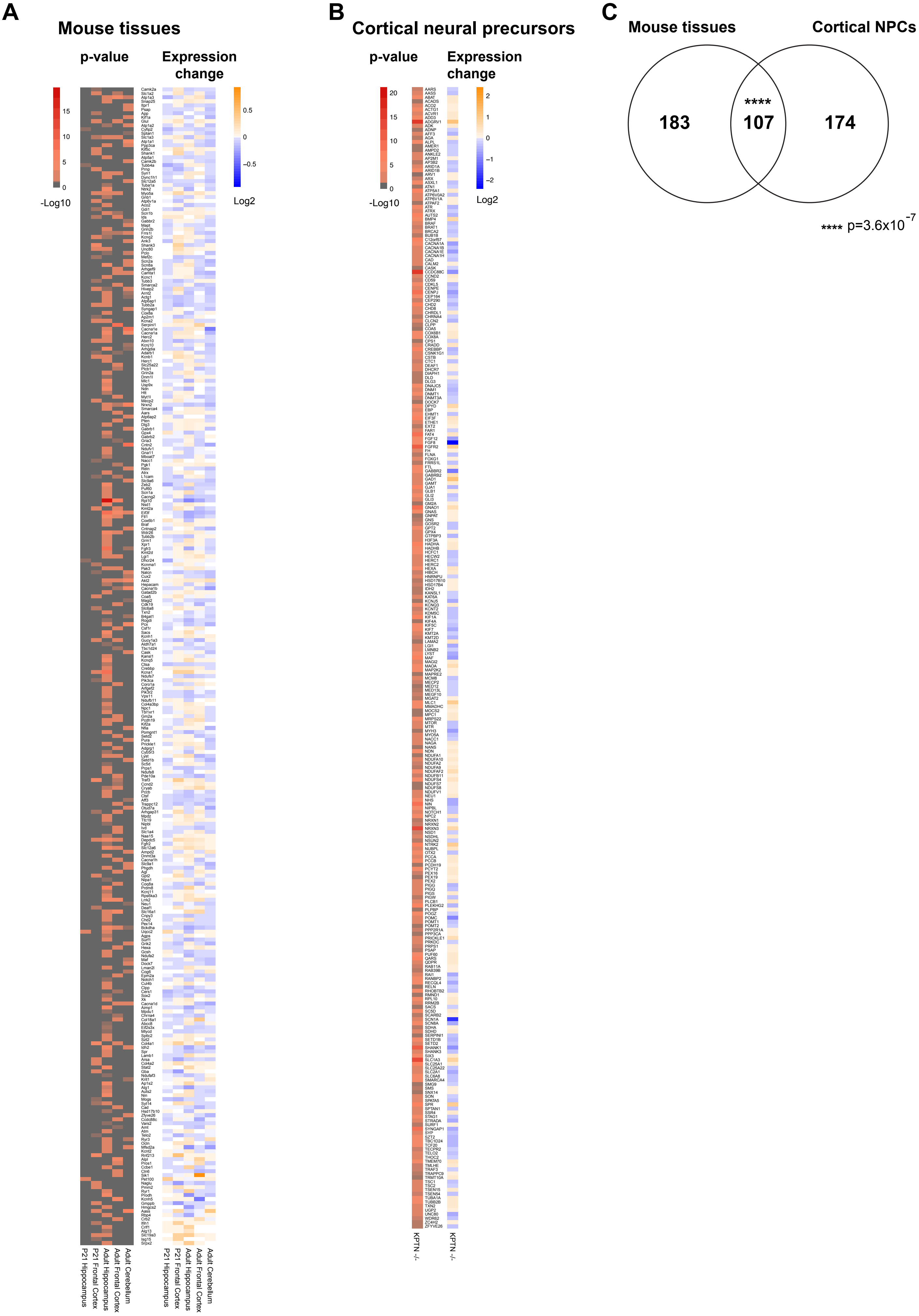

SUPPLEMENTARY FIGURE 8 Expression changes of seizure associated genes in KRD models. Significantly dysregulated genes associated with disorders involving seizures in **A** mouse brain and **B** human NPC models of KRD (statistically significant Log2-fold changes in expression are indicated by non-grey adjusted p-value heatmap cells). **C** Overlap between mouse and human NPC seizure gene signatures (107 genes in common, 1.5 fold over-enriched, $p=3.6x10^{-7}$, hypergeometric test) in KRD models.

Supplementary Table 1

A Mean distance, Wild type

	L2	L3	L4	L5	L6	L7	L8	L9	L11		
L1	4.275	7.495	9.344	8.967	6.560	5.770	5.999	3.456		L1	Bregma
L2		3.283	5.587	6.674	6.187	7.728	9.085	7.682		L2	Lambda
L3			2.890	5.662	7.038	9.727	11.555	10.823		L3	Intersection of interparietal bones with squamous portion of occipital bone at midline
L4				3.781	6.541	9.925	12.119	12.361		L4	Opisthion, midsagittal point on the posterior margin of the foramen magnum
L5					3.662	7.318	9.738	11.201		L5	Caudal most point of basi-occipital bone at mid-sagittal plane
L6						3.659	6.083	8.005		L6	Dorsal-most point of spheno-occipital synchondrosis at mid-sagittal plane
L7							2.455	5.496		L7	Dorsal-most point of inter-sphenoid synchondrosis at mid-sagittal plane
L8								4.248		L8	Rostral end of pre-sphenoid bone at mid-sagittal plane
L9										L9	Caudal-most point of the roof of the olfactory fossa
L10									9.5528	L10	Intersection of the saquamosal suture with temporal crest

B Mean distance, *Kptn -/-*

	L2	L3	L4	L5	L6	L7	L8	L9	L11		
L1	4.235	7.467	9.581	9.183	6.673	6.072	6.262	3.721		L1	Bregma
L2		3.310	5.938	6.986	6.264	7.904	9.220	7.885		L2	Lambda
L3			3.227	5.898	7.000	9.779	11.599	11.005		L3	Intersection of interparietal bones with squamous portion of occipital bone at midline
L4				3.807	6.483	9.963	12.197	12.707		L4	Opisthion, midsagittal point on the posterior margin of the foramen magnum
L5					3.622	7.256	9.707	11.428		L5	Caudal most point of basi-occipital bone at mid-sagittal plane
L6						3.653	6.088	8.142		L6	Dorsal-most point of spheno-occipital synchondrosis at mid-sagittal plane
L7							2.485	5.706		L7	Dorsal-most point of inter-sphenoid synchondrosis at mid-sagittal plane
L8								4.365		L8	Rostral end of pre-sphenoid bone at mid-sagittal plane
L9										L9	Caudal-most point of the roof of the olfactory fossa
L10									9.797	L10	Intersection of the saquamosal suture with temporal crest

C Difference in length, as % of wildtype

	L2	L3	L4	L5	L6	L7	L8	L9	L11		
L1	-0.9	-0.4	2.5	2.4	1.7	5.2	4.4	7.7		L1	Bregma
L2		0.8	6.3	4.7	1.2	2.3	1.5	2.6		L2	Lambda
L3			11.7	4.2	-0.5	0.5	0.4	1.7		L3	Intersection of interparietal bones with squamous portion of occipital bone at midline
L4				0.7	-0.9	0.4	0.6	2.8		L4	Opisthion, midsagittal point on the posterior margin of the foramen magnum
L5					-1.1	-0.9	-0.3	2.0		L5	Caudal most point of basi-occipital bone at mid-sagittal plane
L6						-0.2	0.1	1.7		L6	Dorsal-most point of spheno-occipital synchondrosis at mid-sagittal plane
L7							1.2	3.8		L7	Dorsal-most point of inter-sphenoid synchondrosis at mid-sagittal plane
L8								2.8		L8	Rostral end of pre-sphenoid bone at mid-sagittal plane
L9										L9	Caudal-most point of the roof of the olfactory fossa
L10									2.6	L10	Intersection of the saquamosal suture with temporal crest

D t-test p-values

	L2	L3	L4	L5	L6	L7	L8	L9	L11		
L1	0.6483	0.8313	0.0684	0.034	0.2241	0.0017	0.003	0.0042		L1	Bregma
L2		0.7947	0.0285	0.0055	0.438	0.0686	0.1884	0.0196		L2	Lambda
L3			0.0822	0.0199	0.5744	0.4949	0.6458	0.0825		L3	Intersection of interparietal bones with squamous portion of occipital bone at midline
L4				0.7032	0.2547	0.5696	0.3133	0.0125		L4	Opisthion, midsagittal point on the posterior margin of the foramen magnum
L5					0.4105	0.2673	0.5179	0.0278		L5	Caudal most point of basi-occipital bone at mid-sagittal plane
L6						0.8701	0.9027	0.0428		L6	Dorsal-most point of spheno-occipital synchondrosis at mid-sagittal plane
L7							0.3858	0.0084		L7	Dorsal-most point of inter-sphenoid synchondrosis at mid-sagittal plane
L8								0.0849		L8	Rostral end of pre-sphenoid bone at mid-sagittal plane
L9										L9	Caudal-most point of the roof of the olfactory fossa
L10									0.0356	L10	Intersection of the saquamosal suture with temporal crest

SUPPLEMENTARY TABLE 1 Changes to brain cavity dimensions in KRD model assessed with microcomputed tomography. Inter-landmark distances were calculated (landmarks in Supplementary Figure 3, see Methods) and compared to identify statistically significant differences in mean parameter lengths between male **A** *Kptn*$^{+/+}$ and **B** *Kptn*$^{-/-}$ mice (n=5 each). Statistically significant p-values ($p<0.05$, 2-sample two-tailed Student's t-test) are indicated in green in **D** and boxed in **C**. All lengths are in millimetres.

Supplementary Table 2

Ensembl ID	Gene	P21 Frontal cortex	Hippocampus	Frontal cortex	Cerebellum	Cell types
ENSMUSG00000004891	Nes			13.7%		Radial Glia-like cells
ENSMUSG00000026728	Vim			11.6%		
ENSMUSG00000005360	GLAST/Slc1a3	8.7%	-8%	7.8%		
ENSMUSG00000035686	Thrsp	51.1%	13.5%	38.8%		
ENSMUSG00000027004	Frzb		25.2%			
ENSMUSG00000059325	Hopx			17.8%		
ENSMUSG00000032446	Eomes				61%	Intermediate Progenitors
ENSMUSG00000035033	Tbr1		10%			
ENSMUSG00000038255	Neurod2		16.5%			
ENSMUSG00000020423	Btg2			49.3%		
ENSMUSG00000076431	Sox4		8.9%			

SUPPLEMENTARY TABLE 2 Changes in expression of progenitor markers in KRD mouse model. Differential gene expression analysis between $KPTN^{+/+}$ and $KPTN^{-/-}$ mice for neural stem cell (Radial Glia-like cells) and neurogenic intermediate progenitor (Intermediate Progenitors) markers at both P21 and in adults. Values are expressed as change compared to $KPTN^{+/+}$ wildtype expression levels. All reported changes have adjusted p-values <0.05.

Supplementary Table 3

Phenotypes of haploinsufficient LoF genes dysregulated in *KPTN*$^{-/-}$ NPCs

Gene	ID/DD	Haplo-insufficient LOF	Expression in KPTN-/- NPCs, % of wildtype	Craniofacial phenotypes	Head size	Seizures	Autistic features	Language/speech problems	Brain/nervous system structural abnormalities	References
AUTS2	Y	Y	71.3%	Y	Y (smaller)	Y	Y	Y	+/-	PMID: 23332918, PMID: 27075013
AHDC1	Y	Y	72.4%	Y	+/- (some macro)	+/-	Y	Y	Y	PMID: 24791903, PMID: 27148574
ANKRD11	Y	Y	56.8%	Y	rare (smaller)	+/-	+/-	+/-	Y	PMID: 21782149, PMID: 31191201
CHD7	Y	Y	72.9%	Y	N	N	+/-	(hearing loss)	Y	PMID: 16400610, PMID: 15300250, PMID: 16155193
CREBBP	Y	Y	73.5%	Y	Y (smaller)	+/-	+/-	Y	N	PMID: 12070251, PMID: 18792986, PMID: 26788536
CHD2	Y	Y	73.5%	N	N	Y	+/-	N	+/-	PMID: 23708187, PMID: 25672921
EBF3	Y	Y	53.7%	Y	N	rare	N	Y	N	PMID: 29062322, PMID: 28017373
KMT2D	Y	Y	75.8%	Y	Y (smaller)	Y	+/-	Y	Y	PMID: 21671394, PMID: 21607748, PMID: 15578615
TBR1	Y	Y	71.3%	N	N	N	Y	Y	Y	PMID: 25232744, PMID: 30268909, PMID: 29288087
TCF20	Y	Y	75.2%	Y	Y (some macro)	+/-	Y	Y	+/-	PMID: 30819258
SETD1B	Y	Y	74.9%	Y	N	Y	Y	Y	+/-	PMID: 29322246, PMID: 31110234, PMID: 31685013, PMID: 32546566
ZIC2	Y	Y	71.5%	Y	N	N	N	N	Y	PMID: 9771712, PMID: 11285244, PMID: 19955556

SUPPLEMENTARY TABLE 3 Loss of KPTN function in human neural stem cells results in dysregulation of developmentally important haploinsufficient disorder-associated genes. Twelve chromatin modifying transcriptional regulators, whose heterozygous loss-of-function in humans causes severe developmental disorders, are downregulated by 25-46% in *KPTN*$^{-/-}$ NPCs (*KPTN*$^{-/-}$ expression shown as % of *KPTN*$^{+/+}$ level, adjusted p-values<0.05). Phenotypes associated with loss of function in these disorders are relevant to those seen in KRD patients, including intellectual disability and/or developmental delay (ID/DD), seizures, autistic features, language deficits, and craniofacial dysmorphisms. Their presence was scored from published reports (References) as Present frequently (Y), occasionally (+/-), rarely (rare) or not observed (N).

FILENAME: SUPP FILE1 ANATOMY.xlsx

SUPPLEMENTARY FILE 1 Quantification of neuroanatomical features in the KRD mouse model at P0, P20, and adult stages (16 weeks). Measurements of length or area of 53 features at birth and 78 features in P20 and adult brains were made (See Methods). **TAB 1A** is a list of the 78 neuroanatomical features for brain coronal analysis in P20 and adult stages. **TAB 1B** is the raw measurements for adult male mice at 16 weeks of age across all 78 parameters. **TAB 1C** is the raw measurements for adult female mice at 16 weeks of age across all 78 parameters. **TAB 1D** is the raw measurements for adult male mice at 16 weeks of cellular parameters including cell count, total cell area, cell circularity and cell solidity. **TAB 1E** is a list of the 53 neuroanatomical features for brain coronal analysis in P0 mice. **TAB 1F** is the raw measurements for mice at P0 across all 53 parameters. **TAB 1G** is the raw measurements for male mice at P20 across all 78 parameters. All p-values are from 2-sample two-tailed Student's t-tests.

FILENAME: SUPP FILE2 KRD PROBANDS.xlsx

SUPPLEMENTARY FILE 2 Occipitofrontal circumference measurements of KRD probands and parents. Blue boxes: OFC measurements in centimeters (z-score in brackets) and genotype for previously published and newly identified individuals with KRD from birth to last reported measurement. Green boxes: OFC measurements in centimeters (z-score in brackets) and genotype for heterozygous parents of individuals with KRD. N/K = not known, WT = wild-type. *Progressive chart ID* indicates individuals plotted in Fig. 5B and Supplementary Fig. 5. *Family ID* identifies groups of siblings within nuclear families. *Reference* indicates first publication of individual. See *Materials and methods* for details of z-score calculations.

FILENAME: SUPP FILE3 RNASEQ.xlsx

SUPPLEMENTARY FILE 3 RNA-Seq analysis of differential gene expression in KRD models. TAB A: DESeq2 summary data across all mouse tissues and stages. **TAB B**: DESeq2 summary data from *KPTN*$^{+/-}$ and *KPTN*$^{-/-}$ NPCs. *BaseMean* is normalised mean read count across the tissue for each gene, *log2FoldChange* is the Log2Fold change when comparing LoF model to matched wildtype samples, *padj* is the adjusted p-value output from DESeq2 for the differential expression (see methods for details), *E18* denotes embryonic day 18 brain, *FrC* is prefrontal cortex, *Hipp* is hippocampus, *Cer* is cerebellum, *p21* denotes postnatal day 21, and *adult* denotes adult mice. **TAB C**: List of differentially expressed genes detected in *TSC*$^{-/-}$ neurons from Winden et al 2019**.**

FILENAME: SUPP FILE4 MOUSE PATHWAY ANALYSIS.xlsx

SUPPLEMENTARY FILE 4 Gene set enrichment in mouse KRD model RNA-Seq. Tab A , Summary table of mTOR pathway relevant Gene Ontology enrichment findings for mouse RNA-Seq samples. **Tab B,** Results of hypergeometric test specifically for enrichment of mTOR pathway genes in the KRD model mouse RNA-Seq samples ($\alpha<0.05$). Complete individual Gene Ontology enrichment findings are provided in labelled tabs.

FILENAME: SUPP FILE5 WESTERN BLOTS.pdf

SUPPLEMENTARY FILE 5 Western blots for Kptn mouse model exploring mTOR signaling. All primary images of Western blots for adult brains (page 1), adult hippocampus (page 2), p21 whole brains (page 3) probed with anti-ribosomal protein S6 (upper panel) and the phosphorylated form of the same protein (lower panel). Genotypes are indicated by a one letter code; W = wild-type Kptn+/+;M=LoF Kptn-/-;X=failed sample. Western blot of brains from Rapamycin (WR and MR) and vehicle treated (WV and MV) mice (3 days of treatment) of both genotypes (as above), probed with the antibodies described above (page 4).
